# Development of a wgMLST method for investigating foodborne botulism cases through genome comparisons

**DOI:** 10.3389/fmicb.2026.1920805

**Published:** 2026-10-14

**Authors:** Lori M. Gladney, Jessica L. Halpin, Heather A. Carleton, Hannes Pouseele, Carolina Lúquez

**Affiliations:** 1 Enteric Diseases Laboratory Branch, Division of Foodborne, Waterborne and Environmental Diseases, National Center for Emerging Zoonotic and Infectious Diseases, Centers for Disease Control and Prevention, Atlanta, GA, United States; 2 Biomérieux Benelux, Schaerbeek, Belgium

**Keywords:** botulism, *Clostridium botulinum*, foodborne investigation, high-quality SNP typing, wgMLST

## Abstract

*Clostridium botulinum*, which can cause foodborne botulism through production of botulinum toxin in contaminated foods, is classified as a Tier 1 select agent in the United States. Since the ability to share isolates is federally regulated, approaches to sharing molecular data, including whole genome sequence data (WGS), are needed. We have implemented a whole genome multilocus sequence typing (wgMLST) database for surveillance of Group I *C. botulinum* (serotypes A, B, and F) using WGS to compare strains on a national level during botulism investigations or events. Five-hundred forty-two isolates associated with botulism cases were sequenced and included in the database, including technical replicates. WGS were generated using Illumina (2 x 250 bp MiSeq and 2 x 150 bp MiniSeq) and Ion Torrent (1 x 200 bp PGM and 1 x 400 bp S5) DNA sequencing. Raw read quality, assembly quality, and allele calling metrics were evaluated. wgMLST was compared with High quality SNP-typing (hqSNPs), a currently accepted method, on a subset of five botulism clusters or events. Illumina produced the best quality sequence data (*Q* scores ≥ 30) and the highest number of allele calls. IonTorrent data had quality issues that impacted allele calls and clustering of epidemiologically related isolates. Therefore, sequencing using the Illumina platform will be supported moving forward as part of our national workflow. wgMLST and hqSNPs produced comparable results using Illumina data, and related strains could be distinguished from unrelated strains that were genetically similar and harbored the same toxin subtype. Therefore, a central, wgMLST database will allow U.S. public health laboratories to analyze their own sequencing data and submit data to a national database in PulseNet, the U.S. National molecular surveillance system for foodborne, waterborne, and One Health related outbreaks or events.

## 1 Introduction

*Clostridium botulinum* is a Gram-positive, spore-forming bacterial pathogen that produces a potent neurotoxin responsible for the paralytic disease of botulism. On average, 226 cases of botulism occur each year^1^. *C. botulinum* produces seven well characterized botulinum toxin (BoNT) serotypes (A-G) which are determined by toxin neutralization assays (Sobel, 2005 and Hill et al., 2007). Serotypes can be further characterized by toxin subtypes, which are determined by the *bont* gene amino acid sequences (Peck et al., 2017). At least 41 subtypes have been described: eight subtypes of serotype A (A1–A8), eight subtypes of serotype B (B1–B8), two subtypes of serotype C (C1 and CD), two subtypes of serotype D (D and DC), 12 subtypes of serotype E (E1–E12), and nine subtypes of serotype F (F1–F9) (Peck et al., 2017 and Sikorra et al., 2018). *C. botulinum* is also divided into four metabolic groups designated Groups I, II, III and IV based on biochemical and microbiological traits (Smith et al., 2015). Group I (proteolytic *C. botulinum*) strains produce BoNT types A, B and F and sometimes more than one toxin. *C. botulinum* strains that produce more than one toxin type are denoted as bivalent. Bivalent Group I strains include Ab, Af, Ba, and Bf strains. Some strains carry types A and B *bont* genes but only produce type A toxin and are denoted A(B). Group II (non-proteolytic *C. botulinum*) strains produce BoNT types B, E and F. Group III strains produce BoNT types C, C/D, D and D/C, and Group IV (*C. argentinense*) strains produce BoNT type G. Of these four groups, Group I strains account for most US botulism cases^1^.

Development of tools that can rapidly characterize this important human pathogen are needed in foodborne botulism investigations for purposes of linking clinical cases and for source attribution. In addition, strains of *C. botulinum* type A(B) have been historically difficult to resolve because they present very few genetic differences between each other, and enhanced discrimination of small differences between these strains can be helpful in botulism investigations. This need for enhanced resolution or discrimination of small differences has often required techniques such as analysis of whole genome single nucleotide polymorphisms (SNPs) (Gladney et al., 2021; Hill et al., 2007; Smith et al., 2015; Raphael et al., 2014; Halpin et al., 2017). While whole genome high-quality SNP (hqSNP) typing is useful for identifying botulism clusters, it is not a method that is easily standardized between laboratories and requires a priori knowledge of the genomes to be analyzed since closely related reference genomes are needed for analysis. NCBI Pathogen Detection pipeline is an additional SNP-based tool to compare publicly available genomes, which can be useful (https://www.ncbi.nlm.nih.gov/pathogens/). Other techniques that could be easily standardized include whole genome multilocus sequence typing (wgMLST) or core genome multilocus sequence typing (cgMLST); these methods have been useful for subtyping clusters of enteric pathogens (Katz et al., 2017; Leeper et al., 2023; Joseph et al., 2023 and Leeper et al., 2025). To date, a standardized method for wgMLST of *C. botulinum* has not been described. A scheme for cgMLST of Group I *C. botulinum* using Ridom SeqSphere + software v6.0.0 has been described based on an analysis of 111 genomes (Gonzalez-Escalona and Sharma, 2020). Although Gonzalez-Escalona et al. did not focus on discrimination of botulism clusters, their study reports the overall clustering of *C. botulinum* lineages. cgMLST may be useful when analyses require a core genome approach however, the method requires more evaluation for use in discrimination of additional Groups (II–IV) of *C. botulinum* and cluster analyses.

Other standardized molecular techniques used in the past 25 years to subtype *C. botulinum* isolates include pulsed-field gel electrophoresis (PFGE), seven-gene MLST, multi-loci variable number of tandem repeat analysis, and amplified fragment length polymorphism (AFLP) analysis. These subtyping methods do not provide enough resolution for resolving botulism clusters, and especially *C. botulinum* type A(B) strains (Raphael et al., 2014; Smith et al., 2015; Halpin et al., 2017). wgMLST and cgMLST have become useful and standardized tools for analyzing and resolving clusters of enteric bacteria (Carleton and Gerner-Smidt, 2016; Katz et al., 2017; Gerner-Smidt et al., 2019; Brown et al., 2021; Joseph et al., 2023; Leeper et al., 2023 and Leeper et al., 2025). More importantly, for a variety of enteric bacteria wgMLST and cgMLST approaches have produced comparable results to results obtained with whole genome SNP typing (hqSNPs), without the need for a closely related reference genome.

In this analysis, we report the development of a wgMLST method for investigating foodborne botulism clusters and events through genome comparisons to be used with strains of Group I *C. botulinum*, including highly similar strains of *C. botulinum* type A(B). We also aim to evaluate two sequencing technologies (Illumina and Ion Torrent) and four models of sequencers (MiSeq, MiniSeq, S5 and PGM) and examine clustering of clusters using wgMLST. The availability of a wgMLST database for *C. botulinum* will allow the use of standardized subtyping methods to assess the genetic relationship among isolates from the United States and globally.

## 2 Materials and methods

### 2.1 Development of a wgMLST allele database

Development of a wgMLST allele database was done in coordination with BioMérieux, Inc (BMx). Twenty-six publicly available reference genomes (20 Group I; 6 Group II) (Supplementary Table 1) were used for development of a wgMLST schema for surveillance of *C. botulinum* using the BioNumerics 7.6 platform (Biomérieux, 2022; Joseph et al., 2023 and Leeper et al., 2023). The genomes used to build the wgMLST scheme were selected to ensure representative sequences to cover the commonly seen Group I and II *C. botulinum bont* subtypes at CDC, using complete genomes where available on NCBI at the time the wgMLST scheme was developed. Although the purpose of this study is to evaluate the new wgMLST method for comparison of Group I *C. botulinum* strains, six additional Group II genomes were included in the wgMLST scheme to potentially be able to analyze Group II strains in the future. Mashtree v1.0.4 (Katz et al., 2019) was used to confirm clustering and diversity of input genomes used to build the wgMLST scheme (data not shown).

The 26 annotated, publicly available reference genomes were used to develop the wgMLST scheme (Supplementary Table 1) following methods as described in Joseph et al. (2026). The wgMLST (all loci) scheme includes plasmid loci, botulinum neurotoxin gene loci as well as 7-gene MLST loci (Jacobson et al., 2008). Less than 30 loci were duplicated in the wgMLST scheme, representing the same gene from different references that were not removed after initial scheme refinement. As these represent < 0.2% of the overall scheme they were found not to impact cluster detection as any calls in duplicates would be the same in the query genome. Loci were defined as plasmid loci as described in Leeper et al. (2023). No core scheme was defined, and thus the loci definitions do not include these or accessory loci. Loci names for the wgMLST scheme are included in Supplementary Table 2.

When new entries were added to the database, possible alleles from query genomes were compared to the reference allele database containing the wgMLST loci using both assembly-free and assembly-based approaches. Reference allele numbers were assigned to the query allele if the query allele was an exact match to an existing allele in the reference allele database. A new reference allele could be assigned by the assembly-based workflow if the query allele was not an exact match and if the query allele had start and stop codons, ≤ 100 base pairs (bp) of insertions and deletions (indels), and ≥ 85% nucleotide similarity to the reference allele for the locus. Reference allele sequences used to assign new alleles per locus are included in the wgMLST Reference Alleles Supplementary Data File.

The *C. botulinum* database contains 12,470 loci representing the diversity of *C. botulinum* Types A, B, E, and F (Supplementary Table 2, and Supplementary Data File). The scheme was not further modified upon completion of this study The wgMLST scheme may be publicly accessed at https://github.com/ncezid-biome/pn2.0-mlst-databases.

### 2.2 DNA extraction, sequencing, genome assembly and toxin subtyping

Bacterial strains were grown, and DNA was extracted according to methods described in Halpin et al. (2019a,b). We generated whole genome sequence (WGS) data for 542 isolates using multiple platforms: Ion Torrent (PGM and S5 sequencers) and Illumina (MiSeq and MiniSeq sequencers).

Illumina sequencing libraries were prepared using the Nextera XT Kit (Illumina; San Diego, CA), and sequenced on the Illumina MiSeq (Illumina; San Diego, CA) using 2 × 250 bp v2 chemistry or alternatively by using the Illumina DNA Prep Kit (Illumina; San Diego, CA) and sequenced on the Illumina MiniSeq (Illumina; San Diego, CA) with 2 × 150 bp chemistry.

Ion Torrent sequencing libraries were prepared using the Kapa Biosciences 200 bp Kit (Roche; Wilmington, MA) and sequenced on the Ion Torrent Personal Genome Machine (PGM) (Thermo Fisher; Waltham, MA) or alternatively by using the Kapa Biosciences 400 bp Kit (Roche; Wilmington, MA) and sequenced on the Ion Torrent S5 (Thermo Fisher; Waltham, MA).

Sequence data was submitted to NCBI Bioproject PRJNA428620 (Table 1, Supplementary Table 3). Table 1 lists sequence accession numbers for WGS of cases analyzed as part of this study, and Supplementary Table 3 includes sequence accession numbers for additional isolates analyzed as part of the evaluation of the database. Sixty-six isolates were sequenced using two or more sequencing methods, as technologies became available (Supplementary Table 4): PGM and MiSeq (29 isolates), S5 and MiniSeq (22 isolates), S5 and MiSeq (11 isolates), PGM and S5 (2 isolates), PGM, S5 and MiniSeq (1 isolate), and PGM and MiniSeq (1 isolate).

**Table 1:** *C. botulinum* strains associated with botulism events.

CDC strain no./NCBI accession	Event/cluster ID	Location	Botulism type	Source type	Serotype mouse bioassay	Toxin subtype *WGS*	Case information
CDC21519/ SAMN35027462	1	AK	Foodborne	Stool	A	A1, B5	Clinical sample
CDC21520/ SAMN35027463	1	AK	Foodborne	Food	A	A1, B5	Food sample
CDC22726/ SAMN35027464	2	ID	Foodborne	Stool	A	A1	Clinical sample
CDC22727/ SAMN35027465	2	ID	Foodborne	Food	A	A1	Food sample
CDC43305/ SAMN35027466	3	CT	Foodborne	Stool	A	A1, B5	Clinical sample
CDC43306/ SAMN35027467	3	CT	Foodborne	Food	A	A1, B5	Food sample
CDC28045/ SAMN35027468	4	KY	Foodborne	Food	B	B1	Food sample
CDC28983/ SAMN35027469	4	KY	Foodborne	Stool	B	B1	Clinical sample
CDC60143/ SAMN35027470	5	OH	Foodborne	Stool	A	A1	Case 1 Clinical sample
CDC60148/ SAMN35027471	5	OH	Foodborne	Stool	A	A1	Case 2 Clinical sample
CDC60184/ SAMN35027472	5	OH	Foodborne	Food	A	A1	Food sample
CDC60150/ SAMN35027473	5	OH	Foodborne	Stool	A	A1	Case 3 Clinical sample
CDC60191/ SAMN35027474	5	OH	Foodborne	Stool	A	A1	Case 4 Clinical sample

Genome assembly and quality filtering was performed on raw reads using SPAdes version 3.0.0 for Ion torrent data assembled outside BioNumerics or using SPAdes version 3.7.1 for data within BioNumerics 7.6.3 (Bankevich et al., 2012; Joseph et al., 2023 and Truong et al., 2021). *bont* gene subtypes were determined with CLC Genomics Workbench v10 using the Map to Reference tool (CLC bio, Aarhus, Denmark). Seven-gene Multi-Locus Sequence Typing (MLST) types were determined by querying the *C. botulinum* PubMLST database (Jacobson et al., 2008).

### 2.3 Sequence, assembly, and allele quality assessment

We analyzed 542 sequences generated in house to using the wgMLST database and evaluated overall quality and wgMLST allele calls (Supplementary Table 3). We compared sequences by sequencer type (PGM, S5, MiSeq or MiniSeq) and evaluated the average and median value for metrics identified. Raw read quality was assessed using SneakerNet WGS QA QC Pipeline^2^ including average quality, sequencing coverage, and the average and median for each metric by sequencer type.

wgMLST quality analysis was performed using BioNumerics version 7.6.3. Alleles were identified by two methods: an assembly-free approach using kmers found in raw reads and an assembly-based approach. The settings used for assembly-free allele calling included the following: kmer size 35, minimum coverage 3x and presence of at least 1x coverage of a forward and reverse read. The settings for assembly-based allele calling included 80% percent homology and the identification of required start and stop codon in the assembly. wgMLST quality and summary allele calls were assessed. The BioNumerics quality metrics were gathered for all sequences by sequencer type and the average and median by sequencer type was determined. High quality sequences will be defined by meeting the following metrics: high quality scores (Q30 or higher), average *de novo* coverage (40x or higher), the lowest number of ambiguous base calls, genome length consistent with strains of Group I *C. botulinum* (3.5–4.5 Mb; Moore and Lacey, 2019), unfragmented assemblies (the least number of contigs), the lowest number of multiple allele calls, and the highest number of confirmed and present wgMLST allele calls when compared to sequences generated by other sequencing chemistries. Boxplots were generated in Excel. Isolates whose quality was calculated at zero in the average (median) assessment were excluded from the analysis. Isolates were downsampled to approximately 100–120x using Seqtk^3^ version 1.3 (settings: seed = s100, number of reads = 1,500,000) when necessary for overabundance of coverage before the assessment.

### 2.4 Comparison of wgMLST analysis and Lyve-SET high quality SNP analysis

Genomic clustering data was generated from wgMLST and hqSNP analyses of 13 strains from foodborne botulism cases and food pairs in the United States from two *C. botulinum* type A (*bont*/A1) clusters, two *C. botulinum* type A(B) (*bont*/A1, B5) clusters, and one *C. botulinum* type B (*bont*/B1) cluster. These clusters included subtypes commonly observed in the United States (Table 1). Additional outgroups or sporadic cases were included in the analysis (Supplementary Table 3). The sequences analyzed were generated using either Illumina DNA Prep Library Kit with MiniSeq chemistry (150 bp paired-reads) or Illumina NexteraXT Library Prep Kit with MiSeq v2 chemistry (250 bp paired-reads).

Sequences were analyzed with the wgMLST pipeline in BioNumerics and Lyve-SET High-Quality SNP (hqSNP) Pipeline version 1.1.4f (Katz et al., 2017) following methods described in Gladney et al. (2021) for the hqSNP comparison. Fast matching was used in BioNumerics 7.6.3 (wgMLSTv2 characters, categorical values, < 100 alleles) to identify closest outgroup for inclusion in the wgMLST and hqSNP comparisons. Published, closed, NCBI references for the relevant serotype were used as a reference in the hqSNP analyses.

A Newick tree was constructed from high-quality SNPs identified in two or more genomes (Katz et al., 2017). An unweighted-pair-group-method-with-arithmetic-mean (UPGMA) dendrogram was constructed from wgMLST loci identified in the genomes. Trees were visualized using Mega11 (Tamura et al., 2021). Distance matrices were examined from the output of both tools to establish pairwise allele and SNP ranges for the discrimination of related strains from unrelated strains of the same serotype/toxin subtype.

## 3 Results

### 3.1 Assessment of the wgMLST allele database for *C. botulinum* (Group I and Group II)

The 542 genome sequences were evaluated using the *C. botulinum* database containing 12,470 loci (Supplementary Table 2). These sequences represent 181 different botulism laboratory investigations, including 497 strains from botulism cases and 45 environmental strains representing 15 different *bont* toxin subtypes of serotypes A, B, E and F, including bivalent strains (Supplementary Table 3). Of these 542 sequences, we selected a subset of isolates that were sequenced with two or more sequencing methods (Supplementary Tables 4, 5).

Read quality metrics were assessed and compared for sequences generated by the four sequencing methods. The MiSeq produced the highest read quality for raw sequence data, followed by MiniSeq, S5 and PGM (Table 2). MiSeq read quality metrics were compared to the MiniSeq; both had high average read quality (Q37/Q35 for MiSeq or Q35 for MiniSeq) and combined coverage (100x for MiSeq and 60x for MiniSeq). The quality of the MiSeq reverse read, R2 was generally lower than that of the forward read, R1 (R1 Q37; R2 Q35). The quality of MiniSeq forward and reverse reads were more similar (Q35 for both), this may be due to shorter read lengths generated by the MiniSeq. Overall, MiSeq average and median read quality was higher than the MiniSeq read quality. Ion Torrent S5 reads had sufficient coverage (average 137x or median 105x), but the average read quality was poor (average Q26 and median Q26). PGM sequence data had low coverage (average 39x or median 35x), and low quality scores (average Q28 and median Q28). PGM had higher average and median read quality than the S5. Overall, MiSeq and MiniSeq produced the highest quality raw sequence data.

**Table 2:** Overall read quality metrics by sequencing technology.

Sequencing technology and read pair (where applicable)	Average quality	Coverage
Illumina MiniSeq		
R1 average	35.3	31.8
R1 median	35.9	28.5
R2 average	35.1	31.8
R2 median	35.5	28.6
Illumina MiSeq		
R1 average	37.4	50.4
R1 median	37.5	49.3
R2 average	35.5	50.4
R2 median	35.7	49.8
Ion torrent S5		
Average	26.4	136.6
Median	26.6	105.1
Ion torrent PGM		
Average	28.2	39.4
Median	28.5	35.0

Assembly quality and summary allele call metrics were assessed for each of the four sequencing technologies. MiniSeq data produced the highest results for overall quality and summary allele call metrics, followed by MiSeq, S5 and PGM data (Table 3, Supplementary Figure 1). Two S5 sequences were excluded from the summary and allele call analysis due to their quality being calculated at zero in the average (median) assessment.

**Table 3:** Sequence quality assessment per sequencing platform. Sequence tehnology and (number) of sequenced isolates listed. For quality assessment average (median) are listed.

Sequencing technology (number of isolates)	Average quality	Number of contigs	Average *denovo* coverage	Number of ambiguous bases	Number of loci with multiple Allele calls	Number of confirmed Alleles (two methods)^a^	Number of present Alleles (one method)^b^
MiniSeq sequences (87)	35 (36)	78 (53)	61 (56)	2212 (228)	12 (9)	3148 (3337)	3443 (3457)
MiSeq sequences (107)	37 (37)	162 (66)	100 (100)	24312 (4032)	10 (10)	3157 (3351)	3280 (3436)
S5 sequences (243)^c^	26 (27)	154 (90)	120 (101)	10037 (1842)	38 (31)	2610 (2802)	3218 (3351)
PGM sequences (103)	28 (28)	436 (237)	39 (36)	10694 (5721)	78 (64)	1669 (1708)	2530 (2571)

^a^Total alleles called by both methods (assembly-free and assembly-based allele calls).

^b^Total alleles called by either method (assembly-free or assembly-based allele calls).

^c^Two S5 sequences were removed from the quality assessment based on exclusion criteria.

MiniSeq and MiSeq data had combined average quality scores of 35 and 37 (median 36 and 37) while S5 and PGM had combined average quality scores of 26 and 28 (median 27 and 28). The MiniSeq and MiSeq read quality was higher than S5 and PGM; a 7–10 point difference was typically observed between Illumina (MiniSeq/MiSeq) and Ion Torrent (S5/PGM) read quality (Table 3, Supplementary Table 5, and Supplementary Figure 1). The MiniSeq and MiSeq data had an average of 61 and 100x average *denovo* coverage (median 56 and 100) while S5 and PGM had an average of 120 and 39x (median 101 and 36). MiniSeq, MiSeq, and S5 sequences generally have sufficient coverage (40x or higher). S5 and PGM sequences were of poor quality (typically below Q30); in addition, PGM assemblies on average had less than 40x coverage.

MiniSeq and MiSeq data produced an average of 2,212 and 24,312 ambiguous bases (median 228 and 4,032) while S5 and PGM produced an average of 10,037 and 10,694 ambiguous bases (median 1,842 and 5,721) (Table 3, Supplementary Figure 1). For some sequences, the number of ambiguous bases approached 100,000 and as high as 400,830; these sequences had 16x to 109x *denovo* assembly coverage, and produced poor assemblies due to increased fragmentation and the ambiguous bases may have been the result of differences in library preparation methods used and/or sequence runs overclustering in the case of the Illumina data (high cluster density). High numbers of ambiguous bases in these sequences also reduced the number of allele calls. MiniSeq produced sequence data with fewer ambiguous bases than the MiSeq, S5 and PGM.

The genome assembly size of *C. botulinum* ranged from 3.5 to 4.5 Megabases. Assemblies of MiniSeq and MiSeq data contained an average of 78 and 162 contigs (median 53 and 66) while S5 and PGM assemblies had an average of 154 and 436 contigs (median 90 and 237). The MiniSeq and MiSeq produced high quality assemblies that were more contiguous than the S5 and PGM and were typically less than 100 contigs and had over 50x combined genome coverage (Table 3, Supplementary Figure 1). Nine sequences (three MiSeq and six PGM) produced assemblies with genome sizes that fell below the expected range of 3.5–4.5 Mb for Group I *C. botulinum* (698,975–3,366,482 bases), likely due to coverage (either below 40x or above 1000x), though for one genome, the coverage was 44x and was potentially impacted by poor read quality of Q26–Q29.

MiniSeq and MiSeq data had an average of 12 and 10 multiple allele calls (median 9 and 10) while S5 and PGM had an average of 38 and 78 (median 31 and 64). All sequencing methods generated multiple allele calls at the same locus, defined as different allele number assignments by assembly-free vs. assembly-based allele calling. However, larger numbers of multiple allele calls were observed in Ion Torrent S5 and PGM data likely due to poorer quality (Table 3, Supplementary Figure 1).

MiniSeq and MiSeq data had an average of 3,148 and 3,157 confirmed allele calls (median 3,337 and 3,351) while S5 and PGM had an average of 2,610 and 1,669 (median 2,802 and 1,708). MiniSeq and MiSeq data had an average of 3,443 and 3,280 present allele calls (median 3,457 and 3,436) while S5 and PGM had an average of 3,218 and 2,530 (median 3,351 and 2,571). The total number of confirmed allele calls (alleles called by both assembly-based and assembly free methods) and present allele calls (alleles called either with the assembly-based or assembly free method) were higher with MiniSeq and MiSeq sequence data than S5 or PGM data (Table 3, Supplementary Figure 1). In some cases, there were few “confirmed” alleles—for some sequences alleles were mainly called by a single method (either assembly-based allele calling or assembly-free allele calling). This was typically associated with PGM or S5 data. Overall, sequences generated by the Illumina platforms generated higher numbers of confirmed alleles compared to Ion Torrent. For all sequencing methods, there were sequences with few present allele calls regardless of which method was used; in these cases, the issue was often multi-factorial (poor quality, high ambiguous bases, poor coverage and high number of contigs). When comparing MiniSeq and MiSeq generated data, MiniSeq generated better assemblies with less ambiguous bases, this can also be impacted by differences between library prep methods (MiSeq libraries were generated with NexteraXT, MiniSeq libraries generated with DNA Prep). Ion Torrent platforms generated sequence data of lower quality that resulted in more fragmented assemblies and fewer allele calls.

Furthermore, Group II sequences were generated only on the MiSeq for this study; allele calls were slightly lower for Group II sequences than Group I sequences that were also sequenced on a MiSeq. Group II sequences had an average of 2,609 (median 2,635) confirmed allele calls, and an average of 2,667 (median 2,671) present allele calls where Group I sequences had an average of 3,227 (median 3358) confirmed allele calls and 3,355 (median 3,446) present allele calls. The overall MiSeq dataset including Group I/II had an average of 3,157 (median 3,351) confirmed allele calls and an average of 3,280 (median 3,436) present allele calls (Table 3).

### 3.2 Evaluation of wgMLST for investigation of Group I *C. botulinum* cases

We evaluated results of wgMLST and hqSNP analysis to compare the new wgMLST method to a reference method (hqSNP), and to confirm clustering of food and clinical isolate pairs. Results for foodborne cases are listed in Table 4, including toxin subtypes, seven-gene MLST types, wgMLST and hqSNP results. For clustering information, we report in Table 4 the number of wgMLST alleles (and SNPs) within foodborne cases and between epidemiologically unrelated cases or sporadic isolates of the same toxin subtype. The five botulism events resulted in less than 10 alleles within related cases, and greater than 13 alleles between unrelated cases by wgMLST, and resulted in less than 10 SNPs within related cases and greater than 14 SNPs between unrelated cases by Lyve-SET hqSNP analysis (Table 4). The number of genomic differences between cases varied by the event and toxin subtype. For example, cases of *C. botulinum* A(B) were more clonal due to fewer differences observed between related cases and unrelated outgroups.

**Table 4:** Results of botulism events examined for quality of clustering and observed ranges of SNP/allele differences, including comparison to other closely related sequences in the wgMLST database.

Event/ cluster ID	Botulism type	Number of isolates	Seven gene MLST sequence type (ST)	Toxin subtype	wgMLST Allele differences (within group)	wgMLST Allele differences (to closest outgroup)	hqSNP differences (within group)	hqSNP differences (to closest outgroup)
1^a^	Foodborne: clinical-food pair	2	4	A1, B5	2	7–9	4	11
2^b^	Foodborne: clinical-food pair	2	1	A1	2	86	1	89–93
3^c^	Foodborne: clinical-food pair	2	6	A1, B5	4	6–9	7	7–12
4^d^	Foodborne: clinical-food pair	2	34	B1	3	20–22	9	25–26
5^e^	Foodborne cluster: clinical-food pairs	5	1	A1	0–2	14–15	0–5	14–19

The closest outgroup identified for each food-clinical pair is a single isolate.

^a^*C. botulinum* Type AB (A1, B5) str. CDC67190 (SAMN02664956) was used as reference for cluster 1 SNP analysis. Outgroup isolate: CDC76212.

^b^*C. botulinum* Type A (A1) str. ATCC 3502 (SAMEA1705919) was used as reference for cluster 2 SNP analysis. Outgroup isolate: CDC69120.

^c^*C. botulinum* Type AB (A1, B5) str. CDC69094 (SAMN04262333) was used as reference for cluster 3 SNP analysis. Outgroup isolate: CDC76214.

^d^*C. botulinum* Type B (B1) str. Okra (SAMN02603535) was used as reference for cluster 4 SNP analysis. Note: plasmid was removed from this reference. Outgroup isolate: CDC66190.

^e^*C. botulinum* Type A (A1) str. Hall (SAMN02603534) was used as reference for cluster 5 SNP analysis. Outgroup isolate: CDC76199.

A comparison of wgMLST and hqSNPs for a cluster of of two *C. botulinum* Type B cases in Kentucky, USA (cluster 4) is depicted in Figure 1 for an example. The strains harbor the *bont*/B1 gene. The clinical isolate (CDC28983) and the strain isolated from food the patient consumed (CDC28045) formed a monophyletic cluster in both the wgMLST dendrogram and hqSNP phylogeny and were very closely related (3 alleles different or 9 hqSNPs different) (Figure 1). These two isolates were separated by 20–200 alleles or 25–271 hqSNPs from other unrelated isolates from the United States and Argentina (CDC60050 and CDC66190) that also harbor the *bont*/B1 gene, and clustered separately from the unrelated strains (Figure 1, Supplementary Table 3). Both the wgMLST dendrogram and hqSNP phylogenetic tree showed similar clustering and had similar topologies (Figure 1).

**Figure 1:**
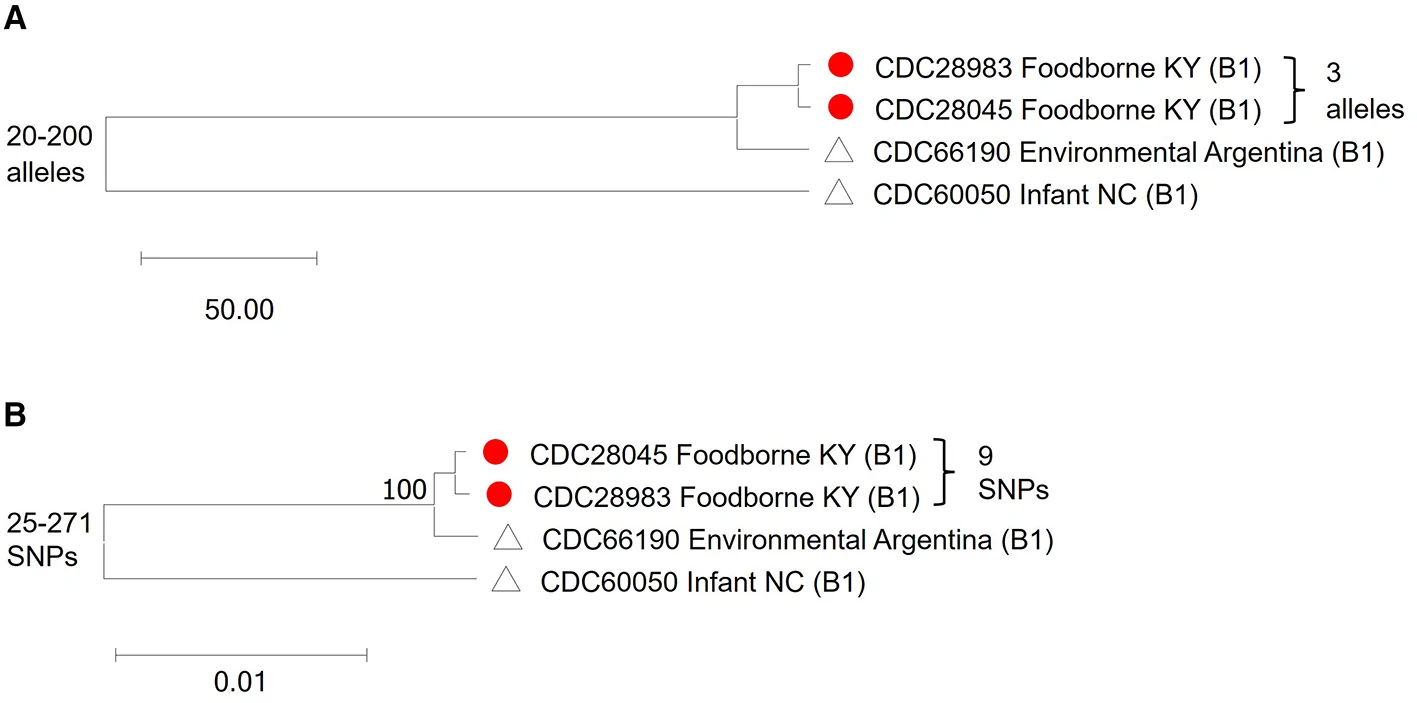
**(A)** Analysis of sequences from a Kentucky Foodborne event (cluster 4) and additional closely related isolates with wgMLST. wgMLST tree of *C. botulinum* type B isolates harboring *bont*/B1 gene. Tree was created in BioNumerics v. 7.6.3 using the unweighted pair group method with arithmetic mean (UPGMA) for similarity clustering of categorical values. The tree includes cluster 4 strains (red circles) and other unpublished sequences from *C. botulinum* type B strains from the CDC reference collection (white triangles). CDC66190 was the closest outgroup strain to the food-clinical pair (CDC28045 and CDC28983) and CDC60050 was an unrelated strain included to help root the tree. Scale bar represents the number of allele differences. **(B)** Analysis of sequences from a Kentucky Foodborne event (cluster 4) and additional closely related isolates with hqSNPs. Maximum-likelihood whole genome high-quality SNP tree of *C. botulinum* type B isolates harboring *bont*/B1 gene. Tree was drawn using Lyve-SET v1.1.4f and includes cluster 4 strains (red circles), and other unpublished sequences from *C. botulinum* type B strains from the CDC reference collection (white triangles). CDC66190 was the closest outgroup strain to the food-clinical pair (CDC28045 and CDC28983) and CDC60050 was an unrelated strain included to help root the tree. Scale bar represents the number of changes per 100 sites. Bootstrap values greater than 70 are shown.

## 4 Discussion

Here we describe the development of a *C. botulinum* wgMLST database for use with botulism case investigations and events. We evaluated two short-read whole genome sequencing chemistries (Ion Torrent and Illumina). Sequences were generated using different sequencers (MiniSeq, MiSeq, S5 and PGM). Overall, the MiniSeq performed the best, followed by MiSeq, S5 and PGM for sequence read, assembly, and wgMLST allele call quality. Overall, per genome the average number of allele calls was higher using Illumina technology, thereby making genomic comparisons using Illumina sequencing generated allele calls more discriminatory. Because adequate allele calling is needed to be able to perform genome comparison, Illumina instruments performed better for use with national molecular surveillance systems for *C. botulinum*. For submission of Group I *C. botulinum* WGS to the PulseNet National Databases, the following criteria will be required: average genome assembly coverage of ≥ 40x, average quality or Q score ≥ 30, and an average genome size of 3,500,000–4,500,000 Mb. A limitation of this study is that not all isolates were sequenced using both sequencing chemistries or all four sequencers. Another limitation of the study is that only short read sequencing technology was evaluated, in the future long read technology will be evaluated as well. Benefits of long read technology include more complete assemblies and potentially more allele calls, and would also be helpful for select agent labs as sequencing in select agent space poses fewer challenges.

Overall, per genome confirmed allele calls were generally greater than 2,500 and present allele calls were generally greater than 3,000; yet the schema encompasses over 12,000 loci. These additional loci in the schema may represent accessory genes, strain specific genes or possibly genes that are exclusive to Group I or II strains as sequences from both groups were used to build the allele database. Additionally, inclusion of Group II genomes in the development of the scheme may have affected the allele calling performance for comparison of Group I genomes, as *C. botulinum* Group I was the primary focus of the study. Group I allele calls were slightly lower than expected as input genomes exhibited 3,499–4,277 genes where the Group I dataset had approximately 3,355 total allele calls. Group II allele calls were the most affected as input genomes exhibited 3,517–3,831 genes where the Group II dataset had approximately 2,667 total allele calls. The inclusion of duplicated loci in the wgMLST scheme could also affect the overall appearance of clusters and differences between isolates since duplicated loci can each receive an allele call for the same sequence. Despite this, the new wgMLST method produced comparable results to other methods used for botulism investigations (e.g., hqSNP) for Group I strains; it remains to be seen if comparable results would be obtained for analysis with Group II strains or if this scheme can be used for analysis of Group II strains. Further characterization of the wgMLST loci and validation of the allele calling workflow is ongoing.

When considering Illumina technology, the higher number of MiniSeq allele calls compared to MiSeq may be explained by the consistent quality of both MiniSeq forward and reverse reads. The quality of the MiSeq reverse read was generally one or two points lower than that of the MiSeq forward read, which was common with older versions (v2) MiSeq sequencing chemistry (Wagner et al., 2021). The difference in quality distribution of the paired reads could affect the performance of allele calling. It is important to note that the read lengths were different between MiSeq (250 bp) and MiniSeq (150 bp), which may lead to the differences in quality between R1 and R2 depending on the method used. Differences in allele calls could also be due to MiSeq data generated with older versions of Illumina library preparation chemistry (e.g., Nextera XT), while the MiniSeq data was generated with newer Illumina library preparation chemistry (e.g., DNA Flex, also now known as DNA Prep). Future plans include a validation with newer Illumina sequencing chemistry.

We characterized five foodborne clusters that represent the most common serotypes observed in the United States from Group I *C. botulinum*. Sequences were examined from two *C. botulinum* type A (*bont*/A1) clusters, two *C. botulinum* type A(B) (*bont*/A1, B5) clusters, and one *C. botulinum* type B (*bont*/B1) cluster. We examined clustering of the five foodborne clusters using wgMLST and hqSNP typing and found that results were comparable. Isolates from epidemiologically linked cases were distinguished from other unrelated isolates by both wgMLST and hqSNP typing. wgMLST provided similar resolution to hqSNPs for resolving botulism clusters and has the added benefit of easy standardization. Given the different methods used to generate the hqSNP/wgMLST trees, similar results suggest that the wgMLST method is comparable to hqSNPs and is useful for comparing relatedness of Group I *C. botulinum* strains during botulism events. Other investigations have also reported comparable resolution of clusters of *C. botulinum* with whole genome high quality SNP typing (Gonzalez-Escalona et al., 2014; Raphael et al., 2014; Williamson et al., 2016, and Gonzalez-Escalona and Sharma, 2020).

A cgMLST scheme has also been proposed for phylogenetic analysis of *C. botulinum* Group I, although a larger dataset is needed to evaluate its resolution power to distinguish botulism clusters of related cases or food-clinical pairs (Gonzalez-Escalona and Sharma, 2020). Research on enteric bacteria have reported a correlation of wgMLST, cgMLST and hqSNP subtyping tools and their usefulness for investigating closely related strains within cluster of *Listeria monocytogenes, Escherichia coli, Salmonella enterica*, and *Campylobacter jejuni* (Katz et al., 2017; Leeper et al., 2023; Joseph et al., 2023, and Leeper et al., 2025). Investigations with *Campylobacter* showed that wgMLST and hqSNP analyses can further discriminate isolates that cluster by cgMLST, and that cgMLST may be helpful initially at the start of an investigation to narrow the scope of the analysis (Joseph et al., 2023).

Another limitation of this study is that the analysis was performed using BioNumerics software, which is no longer supported by the parent company, BioMérieux. This software has since been replaced with a cloud-based platform in PulseNet USA, PulseNet 2.0, which uses the same allele scheme in the present study. While the PulseNet 2.0 platform uses the same allele scheme as the present study, it only performs assembly-based allele calling and incorporates specific improvements to allele calling including (1) the incorporation of hash values for allele identification, (2) an allele-filtering step, which ensures high quality locus classification, and (3) improved time and memory performance of allele calling and filtering. Work to validate *C. botulinum* allele calling and cluster detection in PulseNet 2.0 is ongoing and will be the focus of a future publication for foodborne botulism investigations and infant botulism clusters.

### 4.1 Conclusions

In this analysis, we developed a wgMLST method for *C. botulinum*, creating a foundation for standardized genomic comparison and case investigation, while formal validation and implementation in the current PulseNet 2.0 environment remain ongoing.

The findings and conclusions in this report are those of the authors and do not necessarily represent the official position of the Centers for Disease Control and Prevention.

## Data Availability

The datasets presented in this study can be found in online repositories. The names of the repository/repositories and accession number(s) can be found in the article/Supplementary material.

## Footnotes

1
https://www.cdc.gov/botulism/php/national-botulism-surveillance/

2
https://github.com/lskatz/SneakerNet

3
https://github.com/lh3/seqtk
