# Supplementary material for: Development of a wgMLST method for investigating foodborne botulism cases through genome comparisons
Source: Front Microbiol. 2026 Oct 14;17:1920805. doi: 10.3389/fmicb.2026.1920805 (PMC13713324; doi:10.3389/fmicb.2026.1920805)
Supplement: Supplementary file 1 [file Table_1.docx]

**Supplemental Figure 1.** Boxplots of BioNumerics Quality Assessment Metrics for (A) Average Quality, (B) Number of Contigs, (C) Average Denovo Coverage, (D) Number of Ambiguous Bases, (E) Number of Loci with Multiple Allele Calls, (F) Number of Confirmed Alleles and (G) Number of Present Alleles for 540 sequences.

(A)

(B)

(C)

(D)

(E)

(F)

(G)
