# Supplementary material for: Development of a wgMLST method for investigating foodborne botulism cases through genome comparisons
Source: Front Microbiol. 2026 Oct 14;17:1920805. doi: 10.3389/fmicb.2026.1920805 (PMC13713324; doi:10.3389/fmicb.2026.1920805)
Supplement: Supplementary file 2 [file Table_2.docx]

**Supplemental Table 1.** Publicly available genome sequences used to build the *Clostridium botulinum* wgMLST database.

| **Strain** | **Accession Number(s)** | **Serotype(s)** | **Toxin Subtype(s)** | **Group** | **Genome Size (Megabases)** | **GC content (%)** | **Total Genes** | **Total Proteins** | **Genome Completion Level** |
| --- | --- | --- | --- | --- | --- | --- | --- | --- | --- |
| Hall | NC_009698 | A | A1 | I | 3.8 | 28.2 | 3499 | 3332 | Complete |
| ATCC 3502 | NC_009495, NC_009496 | A | A1 | I | 3.9 | 28.2 | 3786 | 3590 | Complete |
| ATCC 19397 | NC_009697 | A | A1 | I | 3.9 | 28.2 | 3697 | 3561 | Complete |
| CDC297 | CP006907 | A | A1 | I | 3.9 | 28.0 | 3674 | 3608 | Complete |
| Kyoto | NC_012563 | A | A2 | I | 4.2 | 28.2 | 3946 | 3754 | Complete |
| CDC1436 | NZ_CP006908, NZ_CP006909 | A | A2 | I | 4.4 | 28.0 | 4277 | 4078 | Complete |
| Loch Maree | NC_010520, NC_010418 | A | A3 | I | 4.3 | 28.1 | 4045 | 3869 | Complete |
| H04402 065 | NC_017299 | A | A5 | I | 3.9 | 28.2 | 3702 | 3402 | Complete |
| NCTC 2916 | NZ_ABDO02000000 | A(B) | A1, B5 | I | 4.0 | 28.5 | 3877 | 3668 | Contig |
| Af84 | NZ_AOSX01000000, NZ_AOSX01000021 | Af | A2, F4, F5 | I | 4.1 | 27.9 | 3973 | 3779 | Contig |
| Okra | NC_010516, NC_010379 | B | B1 | I | 4.1 | 28.2 | 3937 | 3781 | Complete |
| 111 | NZ_AP014696 | B | B2 | I | 3.9 | 28.3 | 3683 | 3519 | Complete |
| Prevot 594 | NZ_CP006902, NZ_CP006901 | B | B2 | I | 4.3 | 27.8 | 4122 | 3951 | Chromosome with gaps |
| Osaka05 | NZ_BAUF01000000 | B | B2 | I | 4.4 | 27.5 | 4168 | 4015 | Scaffold |
| B2 450 | NZ_JXSU01000000 | B | B2 | I | 4.3 | 27.8 | 4112 | 3912 | Scaffold |
| Bf | NZ_ABDP01000000 | B | B5 | I | 4.2 | 28.2 | 4200 | 4051 | Contig |
| 657 | NC_012658, NC_012654, NC_012657 | Ba | A4, B5 (Ba4) | I | 4.3 | 28.0 | 4144 | 3955 | Complete |
| IBCA10-7060 | NZ_JSCF01000000 | Bf/a | B2, F-A (B2f/a) | I | 4.0 | 27.9 | 3892 | 3625 | Contig |
| Langeland | NC_009699, NC_009700 | F | F1 | I | 4.0 | 28.3 | 3791 | 3641 | Complete |
| 230613 | NC_017297, NC_017298 | F | F1 | I | 4.0 | 28.3 | 3795 | 3367 | Complete |
| Eklund 17B (NRP) | NC_010674, NC_010680 | B | B4 | II | 3.8 | 27.5 | 3517 | 3372 | Complete |
| KAPB-3 | NZ_JQOK01000000 | B | B4 | II | 3.9 | 27.3 | 3509 | 3325 | Scaffold |
| Beluga | NZ_ACSC01000000 | E | E1 | II | 4.0 | 27.4 | 3831 | 3684 | Contig |
| Alaska E43 | NC_010723 | E | E3 | II | 3.7 | 27.4 | 3275 | 3133 | Complete |
| CDC66177 | NZ_ALYJ01000000 | E | E9 | II | 3.9 | 27.2 | 3563 | 3382 | Contig |
| 202F | NZ_CP006903, NZ_CP006904 | F | F6 | II | 3.9 | 27.4 | 3639 | 3466 | Complete |
