# Supplementary material for: Development of a wgMLST method for investigating foodborne botulism cases through genome comparisons
Source: Front Microbiol. 2026 Oct 14;17:1920805. doi: 10.3389/fmicb.2026.1920805 (PMC13713324; doi:10.3389/fmicb.2026.1920805)
Supplement: Supplementary file 4 [file Table_4.docx]

**SUPPLEMENTAL TABLE 5**. BioNumerics Quality Metrics (Average Quality and Average De novo Coverage) for *C. botulinum* strains sequenced with two or more methods. Q score and Coverage value are provided. No data is available for cells highlighted in grey.

| **Strain No./NCBI Accession (if applicable)^a^** | **Sequencer/Library ID** | **PGM** | **S5** | **MiSeq** | **MiniSeq** |
| --- | --- | --- | --- | --- | --- |
| CDC21519 | MiSeq/M347-16-029 |  |  | Q36, 123x |  |
| CDC21519 / SAMN35027462 | MiSeq/M347-17-016 |  |  | Q37, 215x |  |
| CDC21519_1 | S5/SN0054-18-025 |  | Q27, 116x |  |  |
| CDC21519_2 | S5/SN0054-18-025 |  | Q26, 123x |  |  |
| CDC21519_3 | S5/SN0054-18-025 |  | Q27, 213x |  |  |
| CDC21520 | MiSeq/M347-16-029 |  |  | Q35, 115x |  |
| CDC21520 / SAMN35027463 | MiSeq/M347-17-016 |  |  | Q36, 193x |  |
| CDC21520_1 | S5/SN0054-18-028 |  | Q28, 68x |  |  |
| CDC21520_2 | S5/SN0054-18-028 |  | Q28, 101x |  |  |
| CDC21520_3 | S5/SN0054-18-025 |  | Q27, 214x |  |  |
| CDC22726 | MiSeq/M347-17-016 |  |  | Q36, 123x |  |
| CDC22726 / SAMN35027464 | MiSeq/M347-16-029 |  |  | Q37, 193x |  |
| CDC22726 | S5/SN0054-19-001 |  | Q28, 127x |  |  |
| CDC22726_1 | S5/SN0054-18-026 |  | Q28, 78x |  |  |
| CDC22726_2 | S5/SN0054-18-026 |  | Q27, 140x |  |  |
| CDC22727 | MiSeq/M347-17-016 |  |  | Q35, 89x |  |
| CDC22727 / SAMN35027465 | MiSeq/M347-16-029 |  |  | Q36, 175x |  |
| CDC22727_1 | S5/SN0054-19-001 |  | Q28, 155x |  |  |
| CDC22727_2 | S5/SN0054-19-001 |  | Q28, 87x |  |  |
| CDC22727_3 | S5/SN0054-19-001 |  | Q28, 215x |  |  |
| CDC28045 | MiSeq/M3235-16-030 |  |  | Q37, 102x |  |
| CDC28045 / SAMN35027468 | MiSeq/M3235-17-019 |  |  | Q37, 109x |  |
| CDC28045 | S5/SN0054-19-001 |  | Q28, 117x |  |  |
| CDC28045_1 | S5/SN0054-18-026 |  | Q27, 161x |  |  |
| CDC28045_2 | S5/SN0054-18-026 |  | Q27, 203x |  |  |
| CDC28983 | MiSeq/M3235-16-030 |  |  | Q36, 83x |  |
| CDC28983 / SAMN35027469 | MiSeq/M3235-17-019 |  |  | Q37, 105x |  |
| CDC28983 | S5/SN0054-18-025 |  | Q27, 119x |  |  |
| CDC28983 | S5/SN0054-19-001 |  | Q28, 146x |  |  |
| CDC37496 | MiSeq/M347-16-023 |  |  | Q37, 62x |  |
| CDC37496 | MiSeq/M347-17-014 |  |  | Q37, 75x |  |
| CDC37496 / SAMN08330576 | PGM/SN0253 | Q29, 92x |  |  |  |
| CDC37498 | MiSeq/M347-16-023 |  |  | Q36, 73x |  |
| CDC37498 | MiSeq/M347-17-014 |  |  | Q37, 91x |  |
| CDC37498 / SAMN08330577 | PGM/SN0253 | Q29, 73x |  |  |  |
| CDC42747 | MiSeq/M3235-16-030 |  |  | Q36, 111x |  |
| CDC42747 | MiSeq/M3235-17-019 |  |  | Q37, 142x |  |
| CDC42747 | S5/SN0054-18-013 |  | Q26, 90x |  |  |
| CDC42747 | S5/SN0054-18-020 |  | Q26, 129x |  |  |
| CDC42747 | S5/SN0054-18-025 |  | Q27, 293x |  |  |
| CDC43305 | MiSeq/M3235-16-030 |  |  | Q35, 117x |  |
| CDC43305 / SAMN35027466 | MiSeq/M3235-17-019 |  |  | Q36, 98x |  |
| CDC43305 | S5/SN0054-18-013 |  | Q26, 198x |  |  |
| CDC43305 | S5/SN0054-18-020 |  | Q26, 109x |  |  |
| CDC43305 | S5/SN0054-18-025 |  | Q27, 199x |  |  |
| CDC43306 | MiSeq/M3235-16-030 |  |  | Q37, 111x |  |
| CDC43306 / SAMN35027467 | MiSeq/M3235-17-019 |  |  | Q37, 119x |  |
| CDC43306 | S5/SN0054-18-013 |  | Q26, 58x |  |  |
| CDC43306 | S5/SN0054-18-020 |  | Q26, 137x |  |  |
| CDC59470 | MiSeq/M3235-16-030 |  |  | Q37, 132x |  |
| CDC59470 | MiSeq/M3235-17-019 |  |  | Q37, 116x |  |
| CDC59470 | S5/SN0054-18-013 |  | Q26, 426x |  |  |
| CDC59470 | S5/SN0054-18-019 |  | Q27, 129x |  |  |
| CDC59470 | S5/SN0054-18-025 |  | Q27, 175x |  |  |
| CDC59471 | MiSeq/M3235-16-030 |  |  | Q36, 34x |  |
| CDC59471 | S5/SN0054-18-013 |  | Q26, 55x |  |  |
| CDC59471 | S5/SN0054-18-020 |  | Q26, 194x |  |  |
| CDC60001 | MiSeq/M347-16-023 |  |  | Q36, 120x |  |
| CDC60001 | MiSeq/M347-17-014 |  |  | Q37, 121x |  |
| CDC60001 | PGM/SN0253 | Q27, 30x |  |  |  |
| CDC60004 | MiSeq/M347-16-023 |  |  | Q37, 147x |  |
| CDC60004 | MiSeq/M347-17-014 |  |  | Q37, 147x |  |
| CDC60004 | PGM/SN0253 | Q28, 30x |  |  |  |
| CDC60006 | MiSeq/M347-17-014 |  |  | Q36, 75x |  |
| CDC60006_1 | MiSeq/M347-16-023 |  |  | Q36, 59x |  |
| CDC60006_2 | MiSeq/M347-16-023 |  |  | Q36, 130x |  |
| CDC60006_3 | MiSeq/M347-16-023 |  |  | Q37, 243x |  |
| CDC60006_1 | PGM/SN0253 | Q28, 28x |  |  |  |
| CDC60006_2 | PGM/SN0253 | Q28, 29x |  |  |  |
| CDC60006_3 / SAMN08296227 | PGM/SN0253 | Q28, 24x |  |  |  |
| CDC60007 | MiSeq/M347-16-023 |  |  | Q36, 125x |  |
| CDC60007_1 | PGM/SN0253 | Q28, 28x |  |  |  |
| CDC60007_2 | PGM/SN0253 | Q28, 31x |  |  |  |
| CDC60012 | MiSeq/M347-16-023 |  |  | Q35, 85x |  |
| CDC60012 | PGM/SN0253 | Q28, 23x |  |  |  |
| CDC60046 | MiSeq/M347-16-029 |  |  | Q35, 16x |  |
| CDC60046 | PGM/SN0253 | Q28, 29x |  |  |  |
| CDC60050 | MiSeq/M3235-16-026 |  |  | Q34, 36x |  |
| CDC60050 | MiSeq/M3235-16-027 |  |  | Q36, 106x |  |
| CDC60050 | MiSeq/M347-17-014 |  |  | Q37, 111x |  |
| CDC60050_1 | PGM/SN0253 | Q28, 26x |  |  |  |
| CDC60050_2 | PGM/SN0253 | Q28, 40x |  |  |  |
| CDC60062 | MiSeq/M3235-16-026 |  |  | Q33, 64x |  |
| CDC60062 | MiSeq/M3235-16-027 |  |  | Q35, 102x |  |
| CDC60062 | MiSeq/M347-16-027 |  |  | Q36, 66x |  |
| CDC60062_1 | MiSeq/M347-17-014 |  |  | Q36, 69x |  |
| CDC60062_2 | MiSeq/M347-17-014 |  |  | Q36, 103x |  |
| CDC60062_1 | PGM/SN0253 | Q29, 54x |  |  |  |
| CDC60062_2 | PGM/SN0253 | Q29, 69x |  |  |  |
| CDC60066 | MiSeq/M3235-16-024 |  |  | Q37, 18x |  |
| CDC60066_1 | MiSeq/M347-16-027 |  |  | Q37, 148x |  |
| CDC60066_2 | MiSeq/M347-16-027 |  |  | Q37, 157x |  |
| CDC60066_1 | MiSeq/M347-17-014 |  |  | Q37, 149x |  |
| CDC60066_2 | MiSeq/M347-17-014 |  |  | Q37, 152x |  |
| CDC60066_1 / SAMN17730277 | PGM/SN0253 | Q26, 38x |  |  |  |
| CDC60066_2 / SAMN17730278 | PGM/SN0253 | Q27, 33x |  |  |  |
| CDC60072 | MiSeq/M3235-16-024 |  |  | Q37, 20x |  |
| CDC60072 | MiSeq/M347-16-027 |  |  | Q36, 122x |  |
| CDC60072 | MiSeq/M347-17-014 |  |  | Q37, 123x |  |
| CDC60072_1 / SAMN17730279 | PGM/SN0253 | Q26, 26x |  |  |  |
| CDC60072_2 / SAMN17730280 | PGM/SN0253 | Q27, 31x |  |  |  |
| CDC60093 | MiSeq/M3235-16-024 |  |  | Q38, 95x |  |
| CDC60093 | MiSeq/M347-17-014 |  |  | Q37, 79x |  |
| CDC60093 / SAMN17730283 | PGM/SN0253 | Q26, 31x |  |  |  |
| CDC60094 | MiSeq/M347-17-014 |  |  | Q37, 63x |  |
| CDC60094 / SAMN17730284 | PGM/SN0253 | Q27, 26x |  |  |  |
| CDC60099_1 | MiSeq/M3235-16-024 |  |  | Q38, 78x |  |
| CDC60099_2 | MiSeq/M3235-16-024 |  |  | Q38, 150x |  |
| CDC60099_1 | MiSeq/M347-17-015 |  |  | Q37, 68x |  |
| CDC60099_2 | MiSeq/M347-17-015 |  |  | Q37, 148x |  |
| CDC60099_1 | PGM/SN0253 | Q28, 44x |  |  |  |
| CDC60099_2 | PGM/SN0253 | Q28, 45x |  |  |  |
| CDC60101 | MiSeq/M3235-16-024 |  |  | Q38, 42x |  |
| CDC60101 | MiSeq/M347-16-027 |  |  | Q38, 143x |  |
| CDC60101 | MiSeq/M347-17-016 |  |  | Q34, 39x |  |
| CDC60101_1 | MiSeq/M347-17-015 |  |  | Q36, 29x |  |
| CDC60101_2 | MiSeq/M347-17-015 |  |  | Q36, 37x |  |
| CDC60101_1 | PGM/SN0253 | Q28, 52x |  |  |  |
| CDC60101_2 | PGM/SN0253 | Q29, 36x |  |  |  |
| CDC60143 | MiSeq/M347-17-015 |  |  | Q37, 46x |  |
| CDC60143 / SAMN35027470 | MiSeq/M3235-16-024 |  |  | Q38, 56x |  |
| CDC60143_1 / SAMN17850758 | PGM/SN0253 | Q27, 36x |  |  |  |
| CDC60143_2 | PGM/SN0253 | Q27, 44x |  |  |  |
| CDC60148 | MiSeq/M3235-16-024 |  |  | Q38, 62x |  |
| CDC60148 / SAMN35027471 | MiSeq/M347-16-027 |  |  | Q37, 54x |  |
| CDC60148 | MiSeq/M347-17-015 |  |  | Q37, 62x |  |
| CDC60148 / SAMN17850759 | PGM/SN0253 | Q27, 41x |  |  |  |
| CDC60150 / SAMN35027473 | MiSeq/M347-16-027 |  |  | Q37, 60x |  |
| CDC60150 / SAMN17850760 | PGM/SN0253 | Q26, 32x |  |  |  |
| CDC60162 | MiSeq/M347-17-015 |  |  | Q35, 67x |  |
| CDC60162 | PGM/SN0253 | Q27, 33x |  |  |  |
| CDC60184 | MiSeq/M347-16-027 |  |  | Q37, 161x |  |
| CDC60184 / SAMN35027472 | MiSeq/M347-17-016 |  |  | Q37, 167x |  |
| CDC60184 / SAMN17850764 | PGM/SN0253 | Q29, 36x |  |  |  |
| CDC60191 | MiSeq/M347-17-016 |  |  | Q35, 69x |  |
| CDC60191 /SAMN35027474 | MiSeq/M347-16-027 |  |  | Q34, 78x |  |
| CDC60191 / SAMN17850767 | PGM/SN0253 | Q28, 39x |  |  |  |
| CDC60225 / SAMN10922117 | S5/SN0054-17-012 |  | Q25, 79x |  |  |
| CDC60225 | PGM/SN0253-16-002 | Q28, 50x |  |  |  |
| CDC64083 | MiniSeq/MN0344-20-003 |  |  |  | Q36, 59x |
| CDC64083 | S5/SN0054-19-007 |  | Q27, 90x |  |  |
| CDC64136 | MiniSeq/MN0344-20-001 |  |  |  | Q32, 34x |
| CDC64136 | S5/SN0054-20-001 |  | Q26, 63x |  |  |
| CDC64137 | MiniSeq/MN0344-20-001 |  |  |  | Q33, 25x |
| CDC64137 | S5/SN0054-20-001 |  | Q26, 127x |  |  |
| CDC64139 | MiniSeq/MN0344-20-001 |  |  |  | Q32, 29x |
| CDC64139 | S5/SN0054-20-001 |  | Q26, 64x |  |  |
| CDC64140 | MiniSeq/MN0344-20-001 |  |  |  | Q32, 68x |
| CDC64140 | S5/SN0054-20-001 |  | Q26, 84x |  |  |
| CDC64144 | MiniSeq/MN0344-20-001 |  |  |  | Q33, 45x |
| CDC64144 | S5/SN0054-20-001 |  | Q26, 61x |  |  |
| CDC64145 | MiniSeq/MN0344-20-001 |  |  |  | Q32, 54x |
| CDC64145 | S5/SN0054-20-001 |  | Q28, 35x |  |  |
| CDC64167 | MiniSeq/MN0344-20-001 |  |  |  | Q32, 38x |
| CDC64167 | S5/SN0054-20-001 |  | Q27, 61x |  |  |
| CDC64168 | MiniSeq/MN0344-20-001 |  |  |  | Q32, 66 |
| CDC64168 | S5/SN0054-20-001 |  | Q27, 307x |  |  |
| CDC64176 | MiniSeq/MN0344-20-001 |  |  |  | Q32, 91x |
| CDC64176 | S5/SN0054-20-001 |  | Q27, 100x |  |  |
| CDC64186 | MiniSeq/MN0344-20-001 |  |  |  | Q33, 76x |
| CDC64186 | S5/SN0054-20-002 |  | Q27, 59x |  |  |
| CDC64188 | MiniSeq/MN0344-20-001 |  |  |  | Q33, 62x |
| CDC64188 | S5/SN0054-20-002 |  | Q27, 137x |  |  |
| CDC64191 | MiniSeq/MN0344-20-001 |  |  |  | Q32, 96x |
| CDC64191 | S5/SN0054-20-002 |  | Q26, 230x |  |  |
| CDC64194 | MiniSeq/MN0344-20-001 |  |  |  | Q33, 80x |
| CDC64194 | S5/SN0054-20-002 |  | Q26, 49x |  |  |
| CDC64196 | MiniSeq/MN0344-20-001 |  |  |  | Q33, 104x |
| CDC64196 | S5/SN0054-20-002 |  | Q27, 88x |  |  |
| CDC64197 | MiniSeq/MN0344-20-001 |  |  |  | Q33, 135x |
| CDC64197 | S5/SN0054-20-002 |  | Q26, 107x |  |  |
| CDC64242 | MiniSeq/MN0344-20-003 |  |  |  | Q36, 51x |
| CDC64242 | S5/SN0054-19-018 |  | Q28, 105x |  |  |
| CDC68141 | MiniSeq/MN0344-20-001 |  |  |  | Q33, 93x |
| CDC68141 | S5/SN0054-20-002 |  | Q27, 84x |  |  |
| CDC68142 | MiniSeq/MN0344-20-001 |  |  |  | Q33, 90x |
| CDC68142 | S5/SN0054-20-002 |  | Q27, 61x |  |  |
| CDC68144 | MiniSeq/MN0344-20-001 |  |  |  | Q33, 77x |
| CDC68144 | S5/SN0054-20-002 |  | Q27, 157x |  |  |
| CDC68145 | MiniSeq/MN0344-20-001 |  |  |  | Q32, 82x |
| CDC68145 | S5/SN0054-20-002 |  | Q27, 135x |  |  |
| CDC68146 | MiniSeq/MN0344-20-001 |  |  |  | Q33, 105x |
| CDC68146 | MiniSeq/MN0344-20-004 |  |  |  | Q36, 72x |
| CDC68146 | S5/SN0054-20-002 |  | Q27, 65x |  |  |
| CDC68146 | PGM/SN0253 | Q28, 39x |  |  |  |
| CDC68147 | MiniSeq/MN0344-20-001 |  |  |  | Q33, 149x |
| CDC68147 | S5/SN0054-20-002 |  | Q27, 96x |  |  |
| CDC68149 | MiniSeq/MN0344-20-005 |  |  |  | Q36, 52x |
| CDC68149 | PGM/SN0253 | Q29, 25x |  |  |  |
| CDC68154 | S5/SN0054-19-007 |  | Q27, 97x |  |  |
| CDC68154 | PGM/SN0253 | Q29, 51x |  |  |  |
| CDC69035_1 | MiSeq/M3235-16-029 |  |  | Q37, 116x |  |
| CDC69035_2 | MiSeq/M3235-16-029 |  |  | Q37, 178x |  |
| CDC69035_1 | MiSeq/M347-17-015 |  |  | Q36, 115x |  |
| CDC69035_2 | MiSeq/M347-17-015 |  |  | Q36, 169x |  |
| CDC69035 | PGM/SN0253-15-194 | Q29, 83x |  |  |  |
| CDC69080 | MiSeq/M3235-16-029 |  |  | Q37, 122x |  |
| CDC69080 | MiSeq/M347-17-015 |  |  | Q36, 119x |  |
| CDC69080 | PGM/SN0253 | Q26, 25x |  |  |  |
| CDC69080 | PGM/SN0253-15-193 | Q29, 26x |  |  |  |
| CDC69095 | MiSeq/M3235-16-029 |  |  | Q37, 116x |  |
| CDC69095 | MiSeq/M347-17-015 |  |  | Q37, 114x |  |
| CDC69095 | PGM/SN0253 | Q26, 29x |  |  |  |
| CDC69119 | MiSeq/M3235-17-019 |  |  | Q37, 67x |  |
| CDC69119 | MiSeq/M347-17-015 |  |  | Q36, 87x |  |
| CDC69119_1 | MiSeq/M3235-16-029 |  |  | Q37, 74x |  |
| CDC69119_2 | MiSeq/M3235-16-029 |  |  | Q37, 88x |  |
| CDC69119_1 | PGM/SN0253 | Q27, 33x |  |  |  |
| CDC69119_2 | PGM/SN0253 | Q27, 33x |  |  |  |
| CDC69120 | MiSeq/M3235-16-029 |  |  | Q37, 55x |  |
| CDC69120 | MiSeq/M3235-16-030 |  |  | Q36, 100x |  |
| CDC69120_1 | MiSeq/M3235-17-019 |  |  | Q37, 48x |  |
| CDC69120_2 | MiSeq/M3235-17-019 |  |  | Q37, 136x |  |
| CDC69120 | PGM/SN0253 | Q27, 35x |  |  |  |
| CDC69135 | MiSeq/M347-16-028 |  |  | Q36, 87x |  |
| CDC69135 | MiSeq/M3235-17-019 |  |  | Q37, 65x |  |
| CDC69135 | PGM/SN0253 | Q28, 24x |  |  |  |
| CDC69146 | MiSeq/M347-16-028 |  |  | Q36, 69x |  |
| CDC69146 | MiSeq/M3235-16-029 |  |  | Q36, 76x |  |
| CDC69146 | MiSeq/M3235-17-019 |  |  | Q37, 54x |  |
| CDC69146 | MiSeq/M347-17-016 |  |  | Q37, 114x |  |
| CDC69146 | PGM/SN0253-15-195 | Q28, 65x |  |  |  |

^a^Strains sequenced in replicates on the same sequence run are denoted with an underscore and a number for the sequencing replicate (e.g. _1, _2).
