# Supplementary material for: Development of a wgMLST method for investigating foodborne botulism cases through genome comparisons
Source: Front Microbiol. 2026 Oct 14;17:1920805. doi: 10.3389/fmicb.2026.1920805 (PMC13713324; doi:10.3389/fmicb.2026.1920805)
Supplement: Supplementary file 5 [file Table_5.docx]

**SUPPLEMENTAL TABLE 3.** Additional unpublished and published *C. botulinum* sequences included in this study.

| **Strain No./NCBI Accession (if applicable)^a^** | **Sequencer/Library ID** | **Botulism Type** | **Location** | **Toxin Subtype^b^** | |
| --- | --- | --- | --- | --- | --- |
| ATCC 3502/SAMEA1705919 | NCBI | Unknown | Unknown | A1 | |
| CDC21519 | MiSeq/M347-16-029 | Foodborne | AK | A1, B5 | |
| CDC21519 / SAMN35027462 | MiSeq/M347-17-016 | Foodborne | AK | A1, B5 | |
| CDC21519_1 | S5/SN0054-18-025 | Foodborne | AK | A1, B5 | |
| CDC21519_2 | S5/SN0054-18-025 | Foodborne | AK | A1, B5 | |
| CDC21519_3 | S5/SN0054-18-025 | Foodborne | AK | A1, B5 | |
| CDC21520 | MiSeq/M347-16-029 | Foodborne | AK | A1, B5 | |
| CDC21520 / SAMN35027463 | MiSeq/M347-17-016 | Foodborne | AK | A1, B5 | |
| CDC21520_1 | S5/SN0054-18-028 | Foodborne | AK | A1, B5 | |
| CDC21520_2 | S5/SN0054-18-028 | Foodborne | AK | A1, B5 | |
| CDC21520_3 | S5/SN0054-18-025 | Foodborne | AK | A1, B5 | |
| CDC21601 / SAMN10922119 | S5/SN0054-17-013 | Infant | CA | B1 | |
| CDC22634 | S5/SN0054-17-006 | Infant | PA | A1 | |
| CDC22726 | MiSeq/M347-17-016 | Foodborne | ID | A1 | |
| CDC22726 | S5/SN0054-19-001 | Foodborne | ID | A1 | |
| CDC22726 / SAMN35027464 | MiSeq/M347-16-029 | Foodborne | ID | A1 | |
| CDC22726_1 | S5/SN0054-18-026 | Foodborne | ID | A1 | |
| CDC22726_2 | S5/SN0054-18-026 | Foodborne | ID | A1 | |
| CDC22727 | MiSeq/M347-17-016 | Foodborne | ID | A1 | |
| CDC22727 / SAMN35027465 | MiSeq/M347-16-029 | Foodborne | ID | A1 | |
| CDC22727_1 | S5/SN0054-19-001 | Foodborne | ID | A1 | |
| CDC22727_2 | S5/SN0054-19-001 | Foodborne | ID | A1 | |
| CDC22727_3 | S5/SN0054-19-001 | Foodborne | ID | A1 | |
| CDC28012 | PGM/SN0253 | Foodborne | UT | A1, B5 | |
| CDC28045 | MiSeq/M3235-16-030 | Foodborne | KY | B1 | |
| CDC28045 | S5/SN0054-19-001 | Foodborne | KY | B1 | |
| CDC28045 / SAMN35027468 | MiSeq/M3235-17-019 | Foodborne | KY | B1 | |
| CDC28045_1 | S5/SN0054-18-026 | Foodborne | KY | B1 | |
| CDC28045_2 | S5/SN0054-18-026 | Foodborne | KY | B1 | |
| CDC28983 | MiSeq/M3235-16-030 | Foodborne | KY | B1 | |
| CDC28983 | S5/SN0054-18-025 | Foodborne | KY | B1 | |
| CDC28983 | S5/SN0054-19-001 | Foodborne | KY | B1 | |
| CDC28983 / SAMN35027469 | MiSeq/M3235-17-019 | Foodborne | KY | B1 | |
| CDC31747 / SAMN10922102 | S5/SN0054-18-011 | Infant | HI | B5 | |
| CDC31846/ SAMN17597318 | MiniSeq/MN0344-20-004 | Infant | CO | B1 | |
| CDC31851 / SAMN17597319 | MiniSeq/MN0344-20-004 | Infant | CO | NT | |
| CDC31956 / SAMN17597320 | MiniSeq/MN0344-20-004 | NA (environmental) | CO | A1, B5 | |
| CDC31965 / SAMN17597321 | MiniSeq/MN0344-20-004 | NA (environmental) | CO | A1, B5 | |
| CDC31966 / SAMN17597322 | MiniSeq/MN0344-20-004 | NA (environmental) | CO | A1, B5 | |
| CDC31969 / SAMN17597323 | MiniSeq/MN0344-20-004 | NA (environmental) | CO | A1, B5 | |
| CDC31973 / SAMN17597324 | MiniSeq/MN0344-20-004 | NA (environmental) | CO | A1, B5 | |
| CDC31974 / SAMN17597325 | MiniSeq/MN0344-20-004 | NA (environmental) | CO | A1, B5 | |
| CDC31976 / SAMN17597326 | MiniSeq/MN0344-20-004 | NA (environmental) | CO | A1, B5 | |
| CDC32337 | S5/SN0054-017-008 | Infant | DC | A1, B5 | |
| CDC32337 | S5/SN0054-017-008 | Infant | DC | A1, B5 | |
| CDC32356 | S5/SN0054-19-002 | Unknown | GA | B5 | |
| CDC33691 | S5/SN0054-18-011 | Infant | HI | A1, B5 | |
| CDC34293 / SAMN10922103 | S5/SN0054-18-011 | Infant | HI | B5 | |
| CDC35258 | S5/SN0054-19-005 | Infant | DE | B7 | |
| CDC36757 / SAMN10922104 | S5/SN0054-17-013 | Infant | HI | B5 | |
| CDC36910 / SAMN17597327 | MiniSeq/MN0344-20-004 | Infant | CO | A1, B5 | |
| CDC36911 / SAMN17597328 | MiniSeq/MN0344-20-004 | Infant | CO | A1, B5 | |
| CDC37208 / SAMN17597330 | MiniSeq/MN0344-20-004 | NA (environmental) | CO | A1, B5 | |
| CDC37220 / SAMN17597329 | MiniSeq/MN0344-20-004 | NA (environmental) | CO | A1, B5 | |
| CDC37369 / SAMN17597331 | MiniSeq/MN0344-20-004 | NA (environmental) | CO | A1, B5 | |
| CDC37370 / SAMN17597332 | MiniSeq/MN0344-20-004 | NA (environmental) | CO | A1, B5 | |
| CDC37391 / SAMN10922105 | S5/SN0054-17-014 | Infant | HI | B5 | |
| CDC37456 | PGM/SN0253 | Foodborne | AK | A1, B5 | |
| CDC37461 | PGM/SN0253 | Foodborne | AK | A1, B5 | |
| CDC37496 | MiSeq/M347-16-023 | Infant | DC | B7 | |
| CDC37496 | MiSeq/M347-17-014 | Infant | DC | B7 | |
| CDC37496 / SAMN08330576 | PGM/SN0253 | Infant | DC | B7 | |
| CDC37498 | MiSeq/M347-16-023 | Infant | DC | B7 | |
| CDC37498 | MiSeq/M347-17-014 | Infant | DC | B7 | |
| CDC37498 / SAMN08330577 | PGM/SN0253 | Infant | DC | B7 | |
| CDC37513 / SAMN08330578 | PGM/SN0253 | Infant | DC | B7 | |
| CDC38704 | S5/SN0054-19-002 | Unknown | Switzerland | F1 | |
| CDC38839 / SAMN10922106 | S5/SN0054-18-011 | Infant | HI | B5 | |
| CDC39168 / SAMN10922107 | S5/SN0054-18-011 | Infant | HI | B5 | |
| CDC39239 / SAMN17597333 | MiniSeq/MN0344-20-004 | Infant | CO | A1, B5 | |
| CDC39242 / SAMN17597334 | MiniSeq/MN0344-20-004 | Infant | CO | A1, B5 | |
| CDC40176 / SAMN10922108 | S5/SN0054-18-023 | Infant | HI | B5 | |
| CDC41623 / SAMN10922109 | S5/SN0054-18-023 | Infant | HI | B5 | |
| CDC42746 | MiSeq/M3235-16-030 | Foodborne | AK | E1 | |
| CDC42746 | MiSeq/M3235-17-019 | Foodborne | AK | E1 | |
| CDC42747 | MiSeq/M3235-16-030 | Foodborne | AK | E1 | |
| CDC42747 | MiSeq/M3235-17-019 | Foodborne | AK | E1 | |
| CDC42747 | S5/SN0054-18-013 | Foodborne | AK | E1 | |
| CDC42747 | S5/SN0054-18-020 | Foodborne | AK | E1 | |
| CDC42747 | S5/SN0054-18-025 | Foodborne | AK | E1 | |
| CDC43305 | MiSeq/M3235-16-030 | Foodborne | CT | A1, B5 | |
| CDC43305 | S5/SN0054-18-013 | Foodborne | CT | A1, B5 | |
| CDC43305 | S5/SN0054-18-020 | Foodborne | CT | A1, B5 | |
| CDC43305 | S5/SN0054-18-025 | Foodborne | CT | A1, B5 | |
| CDC43305 / SAMN35027466 | MiSeq/M3235-17-019 | Foodborne | CT | A1, B5 | |
| CDC43306 | MiSeq/M3235-16-030 | Foodborne | CT | A1, B5 | |
| CDC43306 | S5/SN0054-18-013 | Foodborne | CT | A1, B5 | |
| CDC43306 | S5/SN0054-18-020 | Foodborne | CT | A1, B5 | |
| CDC43306 / SAMN35027467 | MiSeq/M3235-17-019 | Foodborne | CT | A1, B5 | |
| CDC43321 | S5/SN0054-17-006 | Infant | PA | A1 | |
| CDC44575 | PGM/SN0253-16-006 | Infant | HI | B5 | |
| CDC45459 / SAMN10922110 | S5/SN0054-18-011 | Infant | HI | B5 | |
| CDC47455 / SAMN10922111 | S5/SN0054-18-011 | Infant | HI | B5 | |
| CDC48611 / SAMN10922120 | S5/SN0054-18-011 | Infant | HI | B1 | |
| CDC49917 / SAMN10922112 | S5/SN0054-18-023 | Infant | HI | B5 | |
| CDC53044 / SAMN10922113 | S5/SN0054-18-019 | Infant | HI | B5 | |
| CDC53174 | PGM/SN0253 | Foodborne | Uganda | A2 | |
| CDC5328 | PGM/SN0253 | Wound | MD | A1 | |
| CDC54075 | PGM/SN0253-16-005 | NA (environmental) | Argentina | F5 | |
| CDC54076 | S5/SN0054-19-011 | Infant | Argentina | A2, F4 | |
| CDC54117 | S5/SN0054-17-014 | Infant | HI | A2 | |
| CDC54117 / SAMN10922114 | S5/SN0054-18-023 | Infant | HI | B5 | |
| CDC54250 / SAMN10922115 | S5/SN0054-17-013 | Infant | HI | B5 | |
| CDC55707 | PGM/SN0253 | Infant | NM | A1, B5 | |
| CDC55910 | S5/SN0054-18-027 | Infant | HI | NT | |
| CDC59470 | MiSeq/M3235-16-030 | Foodborne | AK | E3 | |
| CDC59470 | MiSeq/M3235-17-019 | Foodborne | AK | E3 | |
| CDC59470 | S5/SN0054-18-013 | Foodborne | AK | E3 | |
| CDC59470 | S5/SN0054-18-019 | Foodborne | AK | E3 | |
| CDC59470 | S5/SN0054-18-025 | Foodborne | AK | E3 | |
| CDC59471 | MiSeq/M3235-16-030 | Foodborne | AK | E3 | |
| CDC59471 | S5/SN0054-18-013 | Foodborne | AK | E3 | |
| CDC59471 | S5/SN0054-18-020 | Foodborne | AK | E3 | |
| CDC59947 / SAMN10922116 | S5/SN0054-18-027 | Infant | HI | B5 | |
| CDC60001 | MiSeq/M347-16-023 | Infant | MS | A1 | |
| CDC60001 | MiSeq/M347-17-014 | Infant | MS | A1 | |
| CDC60001 | PGM/SN0253 | Infant | MS | A1 | |
| CDC60004 | MiSeq/M347-16-023 | Infant | KY | B1 | |
| CDC60004 | MiSeq/M347-17-014 | Infant | KY | B1 | |
| CDC60004 | PGM/SN0253 | Infant | KY | B1 | |
| CDC60006 | MiSeq/M347-17-014 | Infant | LA | A4, B5 | |
| CDC60006_1 | MiSeq/M347-16-023 | Infant | LA | A4, B5 | |
| CDC60006_1 | PGM/SN0253 | Infant | LA | A4, B5 | |
| CDC60006_2 | MiSeq/M347-16-023 | Infant | LA | A4, B5 | |
| CDC60006_2 | PGM/SN0253 | Infant | LA | A4, B5 | |
| CDC60006_3 | MiSeq/M347-16-023 | Infant | LA | A4, B5 | |
| CDC60006_3 / SAMN08296227 | PGM/SN0253 | Infant | LA | A4, B5 | |
| CDC60007 | MiSeq/M347-16-023 | Infant | NM | B2 | |
| CDC60007_1 | PGM/SN0253 | Infant | NM | B2 | |
| CDC60007_2 | PGM/SN0253 | Infant | NM | B2 | |
| CDC60010 | PGM/SN0253 | Foodborne | NC | A1 | |
| CDC60012 | MiSeq/M347-16-023 | Infant | AZ | NT | |
| CDC60012 | PGM/SN0253 | Infant | AZ | B5 | |
| CDC60044 | PGM/SN0253 | Infant | OK | A1, B5 | |
| CDC60046 | MiSeq/M347-16-029 | Infant | AZ | B5 | |
| CDC60046 | PGM/SN0253 | Infant | AZ | B5 | |
| CDC60050 | MiSeq/M3235-16-026 | Infant | NC | B1 | |
| CDC60050 | MiSeq/M3235-16-027 | Infant | NC | B1 | |
| CDC60050 | MiSeq/M347-17-014 | Infant | NC | B1 | |
| CDC60050_1 | PGM/SN0253 | Infant | NC | B1 | |
| CDC60050_2 | PGM/SN0253 | Infant | NC | B1 | |
| CDC60062 | MiSeq/M3235-16-026 | Infant | LA | B1 | |
| CDC60062 | MiSeq/M3235-16-027 | Infant | LA | B1 | |
| CDC60062 | MiSeq/M347-16-027 | Infant | LA | B1 | |
| CDC60062_1 | MiSeq/M347-17-014 | Infant | LA | B1 | |
| CDC60062_1 | PGM/SN0253 | Infant | LA | B1 | |
| CDC60062_2 | MiSeq/M347-17-014 | Infant | LA | B1 | |
| CDC60062_2 | PGM/SN0253 | Infant | LA | B1 | |
| CDC60066 | MiSeq/M3235-16-024 | Foodborne | Ethiopia | A2 | |
| CDC60066_1 | MiSeq/M347-16-027 | Foodborne | Ethiopia | A2 | |
| CDC60066_1 | MiSeq/M347-17-014 | Foodborne | Ethiopia | A2 | |
| CDC60066_1 / SAMN17730277 | PGM/SN0253 | Foodborne | Ethiopia | A2 | |
| CDC60066_2 | MiSeq/M347-16-027 | Foodborne | Ethiopia | A2 | |
| CDC60066_2 | MiSeq/M347-17-014 | Foodborne | Ethiopia | A2 | |
| CDC60066_2 / SAMN17730278 | PGM/SN0253 | Foodborne | Ethiopia | A2 | |
| CDC60072 | MiSeq/M3235-16-024 | Foodborne | Ethiopia | A2 | |
| CDC60072 | MiSeq/M347-16-027 | Foodborne | Ethiopia | A2 | |
| CDC60072 | MiSeq/M347-17-014 | Foodborne | Ethiopia | A2 | |
| CDC60072_1 / SAMN17730279 | PGM/SN0253 | Foodborne | Ethiopia | A2 | |
| CDC60072_2 / SAMN17730280 | PGM/SN0253 | Foodborne | Ethiopia | A2 | |
| CDC60073_1 / SAMN17730281 | PGM/SN0253 | Foodborne | Ethiopia | A2 | |
| CDC60073_2 / SAMN17730282 | PGM/SN0253 | Foodborne | Ethiopia | NT | |
| CDC60077 | PGM/SN0253 | Infant | NC | B1 | |
| CDC60093 | MiSeq/M3235-16-024 | Foodborne | Ethiopia | A2 | |
| CDC60093 | MiSeq/M347-17-014 | Foodborne | Ethiopia | A2 | |
| CDC60093 / SAMN17730283 | PGM/SN0253 | Foodborne | Ethiopia | A2 | |
| CDC60094 | MiSeq/M347-17-014 | Foodborne | Ethiopia | A2 | |
| CDC60094 / SAMN17730284 | PGM/SN0253 | Foodborne | Ethiopia | A2 | |
| CDC60099_1 | MiSeq/M3235-16-024 | Infant | NC | A1, B5 | |
| CDC60099_1 | MiSeq/M347-17-015 | Infant | NC | A1, B5 | |
| CDC60099_1 | PGM/SN0253 | Infant | NC | A1, B5 | |
| CDC60099_2 | MiSeq/M3235-16-024 | Infant | NC | A1, B5 | |
| CDC60099_2 | MiSeq/M347-17-015 | Infant | NC | A1, B5 | |
| CDC60099_2 | PGM/SN0253 | Infant | NC | A1, B5 | |
| CDC60101 | MiSeq/M3235-16-024 | Unknown (adult) | CA | A1, B5 | |
| CDC60101 | MiSeq/M347-16-027 | Unknown (adult) | CA | A1, B5 | |
| CDC60101 | MiSeq/M347-17-016 | Unknown (adult) | CA | A1, B5 | |
| CDC60101_1 | MiSeq/M347-17-015 | Unknown (adult) | CA | A1, B5 | |
| CDC60101_1 | PGM/SN0253 | Unknown (adult) | CA | A1, B5 | |
| CDC60101_2 | MiSeq/M347-17-015 | Unknown (adult) | CA | A1, B5 | |
| CDC60101_2 | PGM/SN0253 | Unknown (adult) | CA | A1, B5 | |
| CDC60143 | MiSeq/M347-17-015 | Foodborne | OH | A1 | |
| CDC60143 / SAMN35027470 | MiSeq/M3235-16-024 | Foodborne | OH | A1 | |
| CDC60143_1 / SAMN17850758 | PGM/SN0253 | Foodborne | OH | A1 | |
| CDC60143_2 | PGM/SN0253 | Foodborne | OH | A1 | |
| CDC60148 | MiSeq/M3235-16-024 | Foodborne | OH | A1 | |
| CDC60148 | MiSeq/M347-17-015 | Foodborne | OH | A1 | |
| CDC60148 / SAMN17850759 | PGM/SN0253 | Foodborne | OH | A1 | |
| CDC60148 / SAMN35027471 | MiSeq/M347-16-027 | Foodborne | OH | A1 | |
| CDC60150 / SAMN17850760 | PGM/SN0253 | Foodborne | OH | A1 | |
| CDC60150 / SAMN35027473 | MiSeq/M347-16-027 | Foodborne | OH | A1 | |
| CDC60162 | MiSeq/M347-17-015 | Foodborne | OH | A2 | |
| CDC60162 | PGM/SN0253 | Foodborne | OH | A2 | |
| CDC60179 / SAMN17850762 | PGM/SN0253 | Foodborne | OH | A1 | |
| CDC60180 / SAMN17850763 | PGM/SN0253 | Foodborne | OH | A1 | |
| CDC60184 | MiSeq/M347-16-027 | Foodborne | OH | A1 | |
| CDC60184 / SAMN17850764 | PGM/SN0253 | Foodborne | OH | A1 | |
| CDC60184 / SAMN35027472 | MiSeq/M347-17-016 | Foodborne | OH | A1 | |
| CDC60185 / SAMN17850765 | PGM/SN0253 | Foodborne | OH | A1 | |
| CDC60186 / SAMN17850766 | PGM/SN0253 | Foodborne | OH | A1 | |
| CDC60191 | MiSeq/M347-17-016 | Foodborne | OH | A1 | |
| CDC60191 / SAMN17850767 | PGM/SN0253 | Foodborne | OH | A1 | |
| CDC60191 /SAMN35027474 | MiSeq/M347-16-027 | Foodborne | OH | A1 | |
| CDC60193 | MiSeq/M3235-16-029 | Infant | OH | B1 | |
| CDC60193 | MiSeq/M347-17-015 | Infant | OH | B1 | |
| CDC60222 | PGM/SN0253-16-002 | Infant | CO | A1, B5 | |
| CDC60222 | PGM/SN0253-16-003 | Infant | CO | A1, B5 | |
| CDC60225 | PGM/SN0253-16-002 | Infant | HI | B5 | |
| CDC60225 / SAMN10922117 | S5/SN0054-17-012 | Infant | HI | B5 | |
| CDC60229 | PGM/SN0253-16-002 | Infant | VA | B1 | |
| CDC60230_1 | PGM/SN0253-16-002 | Infant | VA | B1 | |
| CDC60230_2 | PGM/SN0253-16-002 | Infant | VA | B1 | |
| CDC60233 | PGM/SN0253-16-002 | Wound | AZ | A1, B5 | |
| CDC60241 | PGM/SN0253-16-003 | Infant | RI | B1 | |
| CDC60245_1 | PGM/SN0253-16-003 | Infant | OK | A1, B5 | |
| CDC60245_2 | PGM/SN0253-16-003 | Infant | OK | A1, B5 | |
| CDC60253_1 | PGM/SN0253-16-001 | Infant | KS | A1 | |
| CDC60253_2 | PGM/SN0253-16-001 | Infant | KS | A1 | |
| CDC60255 | PGM/SN0253-16-001 | Unknown (adult) | FL | A1, B5 | |
| CDC60256 | S5/SN0054-17-007 | Infant | NJ | A1, B5 | |
| CDC60258 | PGM/SN0253-16-001 | Infant | NH | B1 | |
| CDC60265 | PGM/SN0253-15-194 | Infant | OH | B1 | |
| CDC60271_1 | PGM/SN0253-16-003 | Infant | UT | A1 | |
| CDC60271_2 | PGM/SN0253-16-003 | Infant | UT | A1 | |
| CDC60272 | PGM/SN0253-16-003 | Infant | CO | A1, B5 | |
| CDC60272 | PGM/SN0253-16-004 | Infant | CO | A1, B5 | |
| CDC60276_1 | PGM/SN0253-16-004 | Infant | WV | B1 | |
| CDC60276_2 | PGM/SN0253-16-004 | Infant | WV | B1 | |
| CDC60278 | S5/SN0054-18-012 | Infant | CO | A1 | |
| CDC60283 | S5/SN0054-17-007 | Infant | NJ | A1, B5 | |
| CDC60293 | S5/SN0054-18-012 | Infant | NJ | NT | |
| CDC61012 | S5/SN0054-17-003 | Infant | AR | B1 | |
| CDC61012 | S5/SN0054-17-012 | Infant | AK | B1 | |
| CDC61017_1 | S5/SN0054-17-003 | Infant | CO | A1, B5 | |
| CDC61017_2 | S5/SN0054-17-003 | Infant | CO | A1, B5 | |
| CDC61034 | S5/SN0054-17-003 | Wound | HI | B5 | |
| CDC61035 / SAMN10922101 | S5/SN0054-18-012 | Wound | HI | B5 | |
| CDC61040 | S5/SN0054-17-003 | Infant | MS | B1 | |
| CDC61043 | S5/SN0054-17-004 | Infant | AZ | B5 | |
| CDC61044 | S5/SN0054-18-016 | Infant | NC | B1 | |
| CDC61056 | S5/SN0054-017-004 | Infant | NC | B1 | |
| CDC61056 | S5/SN0054-017-004 | Infant | NC | NT | |
| CDC61060 | S5/SN0054-017-004 | Foodborne | OK | A1, B5 | |
| CDC61106_1 / SAMN17857382 | S5/SN0054-18-003 | Foodborne | MS | A1, B5 | |
| CDC61106_2 | S5/SN0054-18-003 | Foodborne | MS | A1, B5 | |
| CDC61108 | S5/SN0054-18-005 | Foodborne | MS | A1, B5 | |
| CDC61108 / SAMN17857383 | S5/SN0054-18-003 | Foodborne | MS | A1, B5 | |
| CDC61111 | S5/SN0054-18-005 | Foodborne | MS | A1, B5 | |
| CDC61111 / SAMN17857384 | S5/SN0054-18-003 | Foodborne | MS | A1, B5 | |
| CDC61112 | S5/SN0054-18-004 | Foodborne | MS | A1, B5 | |
| CDC61112 / SAMN17857385 | S5/SN0054-18-005 | Foodborne | MS | A1, B5 | |
| CDC61113_1 / SAMN17857386 | S5/SN0054-18-004 | Foodborne | MS | A1, B5 | |
| CDC61113_2 | S5/SN0054-18-004 | Foodborne | MS | A1, B5 | |
| CDC61114 / SAMN17857388 | S5/SN0054-18-002 | Foodborne | MS | B2 | |
| CDC61114_1 | S5/SN0054-18-004 | Foodborne | MS | A1, B5 | |
| CDC61114_2 / SAMN17857387 | S5/SN0054-18-004 | Foodborne | MS | A1, B5 | |
| CDC61116 | S5/SN0054-18-002 | Foodborne | MS | A1 | |
| CDC61118 | S5/SN0054-18-006 | Foodborne | MS | A1, B5 | |
| CDC61118 / SAMN17857389 | S5/SN0054-18-002 | Foodborne | MS | A1, B5 | |
| CDC61124 | S5/SN0054-18-028 | Infant | TX | B1 | |
| CDC61126_1 | S5/SN0054-18-006 | Foodborne | MS | A1, B5 | |
| CDC61126_2 / SAMN17857390 | S5/SN0054-18-006 | Foodborne | MS | A1, B5 | |
| CDC61143_1 | S5/SN0054-17-004 | Infant | NJ | B1 | |
| CDC61143_2 | S5/SN0054-17-004 | Infant | NJ | B1 | |
| CDC61145_1 | S5/SN0054-18-002 | Foodborne | MS | A1, B5 | |
| CDC61145_1 | S5/SN0054-18-006 | Foodborne | MS | A1, B5 | |
| CDC61145_2 | S5/SN0054-18-006 | Foodborne | MS | A1, B5 | |
| CDC61145_2 / SAMN17857392 | S5/SN0054-18-002 | Foodborne | MS | B2 | |
| CDC61172 | S5/SN0054-19-001 | Infant | NJ | B1 | |
| CDC61175 | S5/SN0054-19-001 | Infant | MS | NT | |
| CDC61180 | S5/SN0054-18-010 | Infant | DC | B7 | |
| CDC61189 | S5/SN0054-18-007 | Wound | NM | A1 | |
| CDC61195 | S5/SN0054-18-010 | Infant | AZ | B5 | |
| CDC61202 | S5/SN0054-18-012 | Foodborne | FL | A1 | |
| CDC61204 | S5/SN0054-18-007 | Infant | CO | A1 | |
| CDC61215 | S5/SN0054-18-007 | Infant | WV | B1 | |
| CDC61248 | S5/SN0054-18-008 | Infant | NJ | B1 | |
| CDC61249 | S5/SN0054-18-008 | Infant | MS | B1 | |
| CDC61255 | S5/SN0054-18-008 | Infant | CO | A1, B5 | |
| CDC62001 | S5/SN0054-18-008 | Infant | GA | A1, B5 | |
| CDC62002 | S5/SN0054-18-010 | Infant | GA | A1 | |
| CDC62003 | S5/SN0054-18-010 | Infant | RI | B1 | |
| CDC62006 | S5/SN0054-18-010 | Infant | NJ | B1 | |
| CDC62022_1 | S5/SN0054-17-005 | Infant | NC | A1 | |
| CDC62022_2 | S5/SN0054-17-005 | Infant | NC | A1 | |
| CDC62024 | S5/SN0054-17-005 | Infant | AR | B1 | |
| CDC62032 | S5/SN0054-17-006 | Foodborne | TX | B1 | |
| CDC62033 | S5/SN0054-17-006 | Foodborne | TX | B1 | |
| CDC62097 | S5/SN0054-17-006 | Infant | TX | A1, B5 | |
| CDC62114 | S5/SN0054-17-016 | Infant | TX | NT | |
| CDC62114_1 | S5/SN0054-19-003 | Infant | TX | NT | |
| CDC62114_2 | S5/SN0054-19-003 | Infant | TX | NT | |
| CDC62114_3 | S5/SN0054-19-003 | Infant | TX | NT | |
| CDC62114_4 | S5/SN0054-19-003 | Infant | TX | NT | |
| CDC62114_5 | S5/SN0054-19-003 | Infant | TX | NT | |
| CDC62115 | S5/SN0054-17-016 | Infant | TX | A4 | |
| CDC62115 | S5/SN0054-18-009 | Infant | TX | A4 | |
| CDC62115_1 | S5/SN0054-17-016 | Infant | TX | A4 | |
| CDC62115_1 | S5/SN0054-19-004 | Infant | TX | A4 | |
| CDC62115_2 | S5/SN0054-19-004 | Infant | TX | A4 | |
| CDC62115_3 | S5/SN0054-19-004 | Infant | TX | A4 | |
| CDC62115_4 | S5/SN0054-19-004 | Infant | TX | A4 | |
| CDC63002 | S5/SN0054-18-009 | Infant | GA | A1 | |
| CDC63003_1 | S5/SN0054-18-016 | Infant | AZ | A4, B5 | |
| CDC63003_2 | S5/SN0054-18-016 | Infant | AZ | A4, B5 | |
| CDC63015 | S5/SN0054-18-009 | Infant | NJ | B1 | |
| CDC63015 | S5/SN0054-18-019 | Infant | NJ | NT | |
| CDC63015_1 | S5/SN0054-18-013 | Infant | NJ | B1 | |
| CDC63015_2 | S5/SN0054-18-013 | Infant | NJ | NT | |
| CDC63021 | S5/SN0054-18-009 | Infant | AZ | B5 | |
| CDC63023 | S5/SN0054-18-012 | Infant | NJ | B5, F2 | |
| CDC63044_1 | S5/SN0054-18-015 | Infant | NJ | NT | |
| CDC63044_2 | S5/SN0054-18-015 | Infant | NJ | B1 | |
| CDC63044_3 | S5/SN0054-18-015 | Infant | NJ | NT | |
| CDC63076_1 | S5/SN0054-18-027 | Infant | AZ | A1 | |
| CDC63076_2 | S5/SN0054-18-027 | Infant | AZ | A1 | |
| CDC63079 | S5/SN0054-18-027 | Infant | AZ | A1 | |
| CDC63081_1 | S5/SN0054-18-028 | Infant | NC | A1, B5 | |
| CDC63081_2 | S5/SN0054-18-028 | Infant | NC | A1, B5 | |
| CDC63084 | S5/SN0054-18-028 | Infant | AZ | B1 | |
| CDC63105 | S5/SN0054-19-001 | Infant | MO | A1 | |
| CDC63147 | MiniSeq/MN0344-20-005 | Infant | TX | B1 | |
| CDC63153 | MiniSeq/MN0344-20-006 | Infant | NJ | B1 | |
| CDC63153_1 | MiniSeq/MN0344-20-005 | Infant | NJ | NT | |
| CDC63153_2 | MiniSeq/MN0344-20-005 | Infant | NJ | NT | |
| CDC64030 | MiniSeq/MN0344-20-004 | Infant | CO | A1 | |
| CDC64047 | MiniSeq/MN0344-20-004 | Infant | AZ | B5 | |
| CDC64050 | MiniSeq/MN0344-20-004 | Infant | NJ | B1 | |
| CDC64053_1 | MiniSeq/MN0344-20-003 | Infant | NJ | A1, B5 | |
| CDC64053_2 | MiniSeq/MN0344-20-003 | Infant | NJ | A1, B5 | |
| CDC64062 | S5/SN0054-19-008 | Infant | NJ | B1 | |
| CDC64083 | MiniSeq/MN0344-20-003 | Infant | NJ | B1 | |
| CDC64083 | S5/SN0054-19-007 | Infant | NJ | B1 | |
| CDC64086_1 | S5/SN0054-19-008 | Infant | NJ | B1 | |
| CDC64086_2 | S5/SN0054-19-008 | Infant | NJ | B1 | |
| CDC64087_1 | S5/SN0054-19-008 | Infant | CO | NT | |
| CDC64087_2 | S5/SN0054-19-008 | Infant | CO | NT | |
| CDC64103 | S5/SN0054-19-007 | Infant | DC | B7 | |
| CDC64103 | S5/SN0054-19-008 | Infant | DC | B7 | |
| CDC64136 | MiniSeq/MN0344-20-001 | Infant | TX | A1 | |
| CDC64136 | S5/SN0054-20-001 | Infant | TX | A1 | |
| CDC64137 | MiniSeq/MN0344-20-001 | Infant | TX | A1, B5 | |
| CDC64137 | S5/SN0054-20-001 | Infant | TX | A1, B5 | |
| CDC64138 | MiniSeq/MN0344-20-001 | Infant | TX | A1, B5 | |
| CDC64139 | MiniSeq/MN0344-20-001 | Infant | TX | B5, F2 | |
| CDC64139 | S5/SN0054-20-001 | Infant | TX | B5, F2 | |
| CDC64140 | MiniSeq/MN0344-20-001 | Infant | TX | A1, B5 | |
| CDC64140 | S5/SN0054-20-001 | Infant | TX | A1, B5 | |
| CDC64141 | S5/SN0054-19-011 | Wound | TX | A1 | |
| CDC64142 | S5/SN0054-19-011 | Wound | TX | A1, B5 | |
| CDC64143 | S5/SN0054-19-011 | Wound | TX | A1, B5 | |
| CDC64144 | MiniSeq/MN0344-20-001 | Infant | TX | A1, B5 | |
| CDC64144 | S5/SN0054-20-001 | Infant | TX | A1, B5 | |
| CDC64145 | MiniSeq/MN0344-20-001 | Infant | TX | A1, B5 | |
| CDC64145 | S5/SN0054-20-001 | Infant | TX | A1, B5 | |
| CDC64147 | S5/SN0054-19-017 | Infant | TX | A1, B5 | |
| CDC64148 | S5/SN0054-19-017 | Wound | TX | A1, B5 | |
| CDC64149 | S5/SN0054-19-017 | Infant | TX | A1, B5 | |
| CDC64150 | S5/SN0054-19-017 | Infant | TX | A1, B5 | |
| CDC64151 | S5/SN0054-19-017 | Infant | TX | A1, B5 | |
| CDC64152 | S5/SN0054-19-014 | Infant | TX | A2 | |
| CDC64153 | S5/SN0054-19-013 | Infant | TX | A2 | |
| CDC64154 | S5/SN0054-19-013 | Infant | TX | A1, B5 | |
| CDC64155 | S5/SN0054-19-017 | Infant | TX | A1, B5 | |
| CDC64156 | S5/SN0054-19-011 | Infant | TX | A1, B5 | |
| CDC64157 | S5/SN0054-19-018 | Foodborne | TX | A1, B5 | |
| CDC64158 | S5/SN0054-19-011 | Infant | TX | A1, B5 | |
| CDC64159 | S5/SN0054-19-009 | Infant | TX | B1 | |
| CDC64160 | S5/SN0054-19-016 | Infant | TX | B1 | |
| CDC64161 | S5/SN0054-19-016 | Infant | TX | B1 | |
| CDC64162 | S5/SN0054-20-001 | Infant | TX | B5, F2 | |
| CDC64163 | S5/SN0054-19-018 | Infant | TX | B1 | |
| CDC64164 | S5/SN0054-19-018 | Infant | TX | B2 | |
| CDC64165 | S5/SN0054-19-017 | Infant | TX | B1 | |
| CDC64167 | MiniSeq/MN0344-20-001 | Infant | TX | B5 | |
| CDC64167 | S5/SN0054-20-001 | Infant | TX | B5 | |
| CDC64168 | MiniSeq/MN0344-20-001 | Infant | TX | B1 | |
| CDC64168 | S5/SN0054-20-001 | Infant | TX | B1 | |
| CDC64169 | S5/SN0054-19-018 | Infant | TX | B5, F2 | |
| CDC64170 | S5/SN0054-19-017 | Infant | TX | B5, F2 | |
| CDC64171 | S5/SN0054-19-017 | Infant | TX | B2 | |
| CDC64172 | S5/SN0054-19-014 | Unknown | TX | B2 | |
| CDC64173 | S5/SN0054-19-014 | Infant | TX | B2 | |
| CDC64174 | S5/SN0054-19-017 | Infant | TX | B5, F2 | |
| CDC64175 | S5/SN0054-19-014 | Foodborne | TX | B2 | |
| CDC64176 | MiniSeq/MN0344-20-001 | Infant | TX | B1 | |
| CDC64176 | S5/SN0054-20-001 | Infant | TX | B1 | |
| CDC64177 | S5/SN0054-19-017 | Foodborne | TX | B1 | |
| CDC64178 | S5/SN0054-19-013 | Infant | TX | B1 | |
| CDC64179 | S5/SN0054-19-013 | Infant | TX | B2 | |
| CDC64180 | S5/SN0054-19-013 | Foodborne | TX | NT | |
| CDC64181 | S5/SN0054-19-017 | Infant | TX | B1 | |
| CDC64182 | S5/SN0054-19-013 | Infant | TX | B1 | |
| CDC64183 | S5/SN0054-19-009 | Infant | TX | B1 | |
| CDC64184 | S5/SN0054-19-014 | Infant | TX | B5 | |
| CDC64185 | S5/SN0054-19-016 | Infant | TX | B1 | |
| CDC64186 | MiniSeq/MN0344-20-001 | Infant | TX | B5, F2 | |
| CDC64186 | S5/SN0054-20-002 | Infant | TX | B5, F2 | |
| CDC64187 | S5/SN0054-19-016 | Infant | TX | B1 | |
| CDC64188 | MiniSeq/MN0344-20-001 | Infant | TX | B1 | |
| CDC64188 | S5/SN0054-20-002 | Infant | TX | B1 | |
| CDC64189 | S5/SN0054-19-009 | Infant | TX | A4, B5 | |
| CDC64190 | S5/SN0054-19-014 | Infant | TX | B1 | |
| CDC64191 | MiniSeq/MN0344-20-001 | Infant | TX | B5 | |
| CDC64191 | S5/SN0054-20-002 | Infant | TX | B5 | |
| CDC64192 | S5/SN0054-19-009 | Infant | TX | B1 | |
| CDC64193 | S5/SN0054-19-009 | Infant | TX | B1 | |
| CDC64194 | MiniSeq/MN0344-20-001 | Infant | TX | B1 | |
| CDC64194 | S5/SN0054-20-002 | Infant | TX | B1 | |
| CDC64195 | S5/SN0054-19-010 | Infant | TX | B1 | |
| CDC64196 | MiniSeq/MN0344-20-001 | Infant | TX | B5, F2 | |
| CDC64196 | S5/SN0054-20-002 | Infant | TX | B5, F2 | |
| CDC64197 | MiniSeq/MN0344-20-001 | Infant | TX | B1 | |
| CDC64197 | S5/SN0054-20-002 | Infant | TX | B1 | |
| CDC64208_1 | MiniSeq/MN0344-20-005 | Infant | OK | NT | |
| CDC64208_2 | MiniSeq/MN0344-20-005 | Infant | OK | A2 | |
| CDC64213 | S5/SN0054-19-018 | Foodborne | CO | A1, B5 | |
| CDC64216 | MiniSeq/MN0344-20-007 | Foodborne | CO | A1, B5 | |
| CDC64218 | MiniSeq/MN0344-20-006 | Foodborne | CO | A1, B5 | |
| CDC64223 | MiniSeq/MN0344-20-007 | Foodborne | CO | A1,B5 | |
| CDC64224 | MiniSeq/MN0344-20-003 | Infant | MD | NT | |
| CDC64225 | MiniSeq/MN0344-20-003 | Wound | MD | B1 | |
| CDC64225 | MiniSeq/MN0344-20-006 | Wound | MD | B1 | |
| CDC64228 | MiniSeq/MN0344-20-003 | Foodborne | MS | NT | |
| CDC64235 | MiniSeq/MN0344-20-003 | Infant | IL | A1, B5 | |
| CDC64237 | S5/SN0054-19-018 | Infant | MO | B1 | |
| CDC64242 | MiniSeq/MN0344-20-003 | Infant | SC | B1 | |
| CDC64242 | S5/SN0054-19-018 | Infant | SC | B1 | |
| CDC64251_1 | MiniSeq/MN0344-20-003 | Infant | IL | A4, B5 | |
| CDC64251_2 | MiniSeq/MN0344-20-003 | Infant | IL | A4, B5 | |
| CDC64262 | MiniSeq/MN0344-20-003 | Infant | NJ | B1 | |
| CDC64289 | MiniSeq/MN0344-20-003 | Infant | MD | B5, F2 | |
| CDC64294 | MiniSeq/MN0344-20-003 | Infant | NJ | B1 | |
| CDC64295 | MiniSeq/MN0344-20-005 | Infant | AR | B1 | |
| CDC64296 | MiniSeq/MN0344-20-005 | Infant | IL | B1 | |
| CDC65069 / SAMN10922118 | S5/SN0054-17-012 | Infant | HI | B5 | |
| CDC66008 / SAMN09791861 | S5/SN0054-017-004 | Foodborne | Thailand | B8 | |
| CDC66089 | PGM/SN0253 | Foodborne | NY | A1, B5 | |
| CDC66144 | MiniSeq/MN0344-20-005 | NA (environmental) | Argentina | A2 | |
| CDC66145 | MiniSeq/MN0344-20-005 | NA (environmental) | Argentina | A1 | |
| CDC66146 | MiniSeq/MN0344-20-006 | NA (environmental) | Argentina | A1 | |
| CDC66147 | MiniSeq/MN0344-20-006 | NA (environmental) | Argentina | A1 | |
| CDC66148 | MiniSeq/MN0344-20-006 | NA (environmental) | Argentina | A2 | |
| CDC66190 | MiniSeq/MN0344-22-007 | NA (environmental) | Argentina | B1 | |
| CDC66197 | S5/SN0054-017-008 | Infant | NJ | A1 | |
| CDC67190/SAMN02664956 | NCBI | Foodborne | AZ | A1, B5 | |
| CDC68125 | MiSeq/M3235-16-029 | Infant | Argentina | B1 | |
| CDC68125 | MiSeq/M347-17-015 | Infant | Argentina | B1 | |
| CDC68130 | S5/SN0054-19-002 | Infant | Argentina | A2, F5 | |
| CDC68131 | S5/SN0054-19-002 | Infant | Argentina | F4 | |
| CDC68132 | S5/SN0054-19-002 | NA (environmental) | Argentina | F4 | |
| CDC68133 | S5/SN0054-19-002 | NA (environmental) | Argentina | A2, F5 | |
| CDC68136 | S5/SN0054-19-002 | Foodborne | Argentina | A2, F5 | |
| CDC68137 | S5/SN0054-19-002 | NA (environmental) | Argentina | B2, F4 | |
| CDC68138 | S5/SN0054-19-002 | NA (environmental) | Argentina | B2, F4 | |
| CDC68139 | S5/SN0054-19-002 | NA (environmental) | Argentina | A2, F5 | |
| CDC68140 | S5/SN0054-19-002 | NA (environmental) | Argentina | A2, F4 | |
| CDC68141 | MiniSeq/MN0344-20-001 | NA (environmental) | Argentina | A2, F5 | |
| CDC68141 | S5/SN0054-20-002 | NA (environmental) | Argentina | A2, F5 | |
| CDC68142 | MiniSeq/MN0344-20-001 | NA (environmental) | Argentina | A2, F5 | |
| CDC68142 | S5/SN0054-20-002 | NA (environmental) | Argentina | A2, F5 | |
| CDC68143 | S5/SN0054-19-010 | NA (environmental) | Argentina | A2, F5 | |
| CDC68144 | MiniSeq/MN0344-20-001 | NA (environmental) | Argentina | A2,F4, F5 | |
| CDC68144 | S5/SN0054-20-002 | NA (environmental) | Argentina | A2, F4,F5 | |
| CDC68145 | MiniSeq/MN0344-20-001 | NA (environmental) | Argentina | A2, F4 | |
| CDC68145 | S5/SN0054-20-002 | NA (environmental) | Argentina | A2, F4 | |
| CDC68146 | MiniSeq/MN0344-20-001 | NA (environmental) | Argentina | A2, F5 | |
| CDC68146 | MiniSeq/MN0344-20-004 | NA (environmental) | Argentina | A2, F5 | |
| CDC68146 | PGM/SN0253 | NA (environmental) | Argentina | A2 | |
| CDC68146 | S5/SN0054-20-002 | NA (environmental) | Argentina | A2, F5 | |
| CDC68147 | MiniSeq/MN0344-20-001 | NA (environmental) | Argentina | A2, F5 | |
| CDC68147 | S5/SN0054-20-002 | NA (environmental) | Argentina | A2, F5 | |
| CDC68149 | MiniSeq/MN0344-20-005 | NA (environmental) | Argentina | A2 | |
| CDC68149 | PGM/SN0253 | NA (environmental) | Argentina | A2 | |
| CDC68150 | MiniSeq/MN0344-20-005 | NA (environmental) | Argentina | A2 | |
| CDC68152 | S5/SN0054-19-007 | NA (environmental) | Argentina | A2 | |
| CDC68153 | S5/SN0054-19-007 | NA (environmental) | Argentina | B2 | |
| CDC68154 | PGM/SN0253 | NA (environmental) | Argentina | B2 | |
| CDC68154 | S5/SN0054-19-007 | NA (environmental) | Argentina | B2 | |
| CDC69011 | PGM/SN0253-15-192 | Infant | CO | B2 | |
| CDC69023_1 | PGM/SN0253-15-192 | Infant | CO | A1, B5 | |
| CDC69023_2 | PGM/SN0253-15-192 | Infant | CO | A1, B5 | |
| CDC69035 | PGM/SN0253-15-194 | Infant | TN | A1, B5 | |
| CDC69035_1 | MiSeq/M3235-16-029 | Infant | TN | A1, B5 | |
| CDC69035_1 | MiSeq/M347-17-015 | Infant | TN | A1, B5 | |
| CDC69035_2 | MiSeq/M3235-16-029 | Infant | TN | A1, B5 | |
| CDC69035_2 | MiSeq/M347-17-015 | Infant | TN | A1, B5 | |
| CDC69043 | PGM/SN0253-16-004 | Foodborne | CO | A4, B5 | |
| CDC69043_1 / SAMN08297101 | PGM/SN0253-15-192 | Foodborne | CO | A4, B5 | |
| CDC69043_2 | PGM/SN0253-15-192 | Foodborne | CO | A4, B5 | |
| CDC69057 | MiSeq/M3235-16-029 | Infant | TX | B5, F2 | |
| CDC69057 | MiSeq/M347-17-015 | Infant | TX | B5, F2 | |
| CDC69060 | MiSeq/M3235-16-029 | Infant | AZ | A1 | |
| CDC69060 | MiSeq/M347-17-015 | Infant | AZ | A1 | |
| CDC69066 | PGM/SN0253-15-193 | Infant | NJ | B1 | |
| CDC69068 / SAMN08354338 | PGM/SN0253-15-193 | Infant | NJ | B7 | |
| CDC69077 | PGM/SN0253-15-193 | Infant | DC | B1 | |
| CDC69080 | MiSeq/M3235-16-029 | Infant | GA | A1, B5 | |
| CDC69080 | MiSeq/M347-17-015 | Infant | GA | A1, B5 | |
| CDC69080 | PGM/SN0253 | Infant | GA | A1, B5 | |
| CDC69080 | PGM/SN0253-15-193 | Infant | GA | A1, B5 | |
| CDC69084 | PGM/SN0253-15-193 | Infant | NJ | B1 | |
| CDC69094/SAMN04262333 | NCBI | Infant | UT | A1, B5 | |
| CDC69095 | MiSeq/M3235-16-029 | Foodborne | OH | B5 | |
| CDC69095 | MiSeq/M347-17-015 | Foodborne | OH | B5 | |
| CDC69095 | PGM/SN0253 | Foodborne | OH | B5 | |
| CDC69111_1 | PGM/SN0253-16-004 | Infant | UT | B2 | |
| CDC69111_2 | PGM/SN0253-16-004 | Infant | UT | B2 | |
| CDC69118_1 | PGM/SN0253 | Infant | VT | A1, B5 | |
| CDC69118_2 | PGM/SN0253 | Infant | VT | A1, B5 | |
| CDC69119 | MiSeq/M3235-17-019 | Infant | NJ | B2 | |
| CDC69119 | MiSeq/M347-17-015 | Infant | NJ | B2 | |
| CDC69119_1 | MiSeq/M3235-16-029 | Infant | NJ | B2 | |
| CDC69119_1 | PGM/SN0253 | Infant | NJ | B2 | |
| CDC69119_2 | MiSeq/M3235-16-029 | Infant | NJ | B2 | |
| CDC69119_2 | PGM/SN0253 | Infant | NJ | B2 | |
| CDC69120 | MiSeq/M3235-16-029 | Infant | CO | A1 | |
| CDC69120 | MiSeq/M3235-16-030 | Infant | CO | A1 | |
| CDC69120 | PGM/SN0253 | Infant | CO | A1 | |
| CDC69120_1 | MiSeq/M3235-17-019 | Infant | CO | A1 | |
| CDC69120_2 | MiSeq/M3235-17-019 | Infant | CO | A1 | |
| CDC69125 | PGM/SN0253-16-004 | Infant | CO | A1 | |
| CDC69135 | MiSeq/M3235-17-019 | Infant | NJ | B1 | |
| CDC69135 | MiSeq/M347-16-028 | Infant | NJ | B1 | |
| CDC69135 | PGM/SN0253 | Infant | NJ | B1 | |
| CDC69146 | MiSeq/M3235-16-029 | Infant | CO | B1 | |
| CDC69146 | MiSeq/M3235-17-019 | Infant | CO | B1 | |
| CDC69146 | MiSeq/M347-16-028 | Infant | CO | B1 | |
| CDC69146 | MiSeq/M347-17-016 | Infant | CO | B1 | |
| CDC69146 | PGM/SN0253-15-195 | Infant | CO | B1 | |
| CDC69157 | PGM/SN0253 | Infant | GA | B1 | |
| CDC75001 | MiniSeq/MN0344-20-003 | Infant | MO | B1 | |
| CDC75005 | MiniSeq/MN0344-20-003 | Foodborne | TX | A1, B5 | |
| CDC75008 | MiniSeq/MN0344-20-003 | Infant | FL | A1, B5 | |
| CDC75010 | MiniSeq/MN0344-20-003 | Infant | NC | B1 | |
| CDC75035 | MiniSeq/MN0344-20-003 | Infant | NC | B7 | |
| CDC75038 | MiniSeq/MN0344-20-003 | Infant | SC | A1, B5 | |
| CDC75040 | MiniSeq/MN0344-20-006 | Infant | MO | A1 | |
| CDC75041 | MiniSeq/MN0344-20-003 | Infant | MD | B1 | |
| CDC75044 | MiniSeq/MN0344-20-003 | Infant | NJ | B1 | |
| CDC75046 | MiniSeq/MN0344-20-003 | Infant | AZ | B5 | |
| CDC75052 | MiniSeq/MN0344-20-007 | Infant | NV | NT | |
| CDC75058 | MiniSeq/MN0344-20-006 | Infant | MD | B1 | |
| CDC76199 | MiniSeq/MN0344-21-017 | Infant | FL | A1 | |
| CDC76212 | MiniSeq/MN0344-21-017 | Foodborne | SC | A1, B5 | |
| CDC76214 | MiniSeq/MN0344-21-017 | Infant | WV | A1, B5 | |
| Hall-A1/SAMN02603534 | NCBI | Foodborne | CA | A1 |  |
| Okra/SAMN02603535 | NCBI | Foodborne | TN | B1 | |
| Okra_1 | PGM/SN0253 | Foodborne | Unknown | B1 | |
| Okra_2 | PGM/SN0253 | Foodborne | Unknown | B1 | |
| Okra_3 | PGM/SN0253 | Foodborne | Unknown | B1 | |

^a^Strains sequenced in replicates on the same sequence run are denoted with an underscore and a number for the sequencing replicate (e.g. _1, _2).

^b^NT refers to a Non-Toxic strain.
